# High-efficiency tandem DSSCs based on tailored naphthalene sensitizers for indoor DSSC efficiency above 25%

**DOI:** 10.1038/s41598-025-30854-0

**Published:** 2025-12-18

**Authors:** Mohamed R. Elmorsy, Samar M. Mohammed, Basant A. Mohamed, Ahmed H. Moustafa, Safa A. Badawy

**Affiliations:** 1 Department of Chemistry, Faculty of Science, Mansoura University, Mansoura, 35516 Egypt; 2 Chemistry Department, Faculty of Science, Zagazig University, Zagazig, 44519 Egypt

**Keywords:** Organic D–π–A sensitizers, Naphthalene donor, Tandem solar cells, Indoor photovoltaics, Photostability, Power conversion efficiency (PCE), Chemistry, Energy science and technology, Engineering, Materials science

## Abstract

Dye-sensitized solar cells (DSSCs) are among the most promising photovoltaic technologies for both outdoor and indoor energy harvesting due to their low cost and spectral versatility. In this work, a new series of organic donor–π–acceptor (D–π–A) sensitizers (**BAM-1–BAM-4**) featuring a naphthalene donor and a phenyl-pyrazole π-bridge were designed to explore how structural variations in electron-withdrawing acceptors influence light absorption, charge transfer, and device efficiency. The dyes were systematically investigated using UV–Vis spectroscopy, electrochemical analysis, and density functional theory calculations, and were applied individually and jointly with the benchmark Black dye in single and parallel tandem DSSCs (PT-DSSCs). BAM-3 and BAM-4 exhibited strong intramolecular charge transfer, red-shifted absorption, and efficient electron injection, achieving power conversion efficiencies of 9.85% and 8.89%, respectively, when co-sensitized with Black dye. The optimized PT-DSSC employing BAM-3 + BAM-4 as the bottom photoanode and Black dye as the top achieved a remarkable 12.13% efficiency under AM 1.5G and 25.85% under 1000 lx indoor illumination, maintaining 95% stability after 300 h of continuous operation. These results demonstrate that molecular engineering of D–π–A dyes combined with tandem co-sensitization provides an effective pathway to achieve high-efficiency, stable DSSCs suitable for both solar and ambient-light applications.

**Supplementary Information:**

The online version contains supplementary material available at 10.1038/s41598-025-30854-0.

## Introduction

**Fig. 1:**
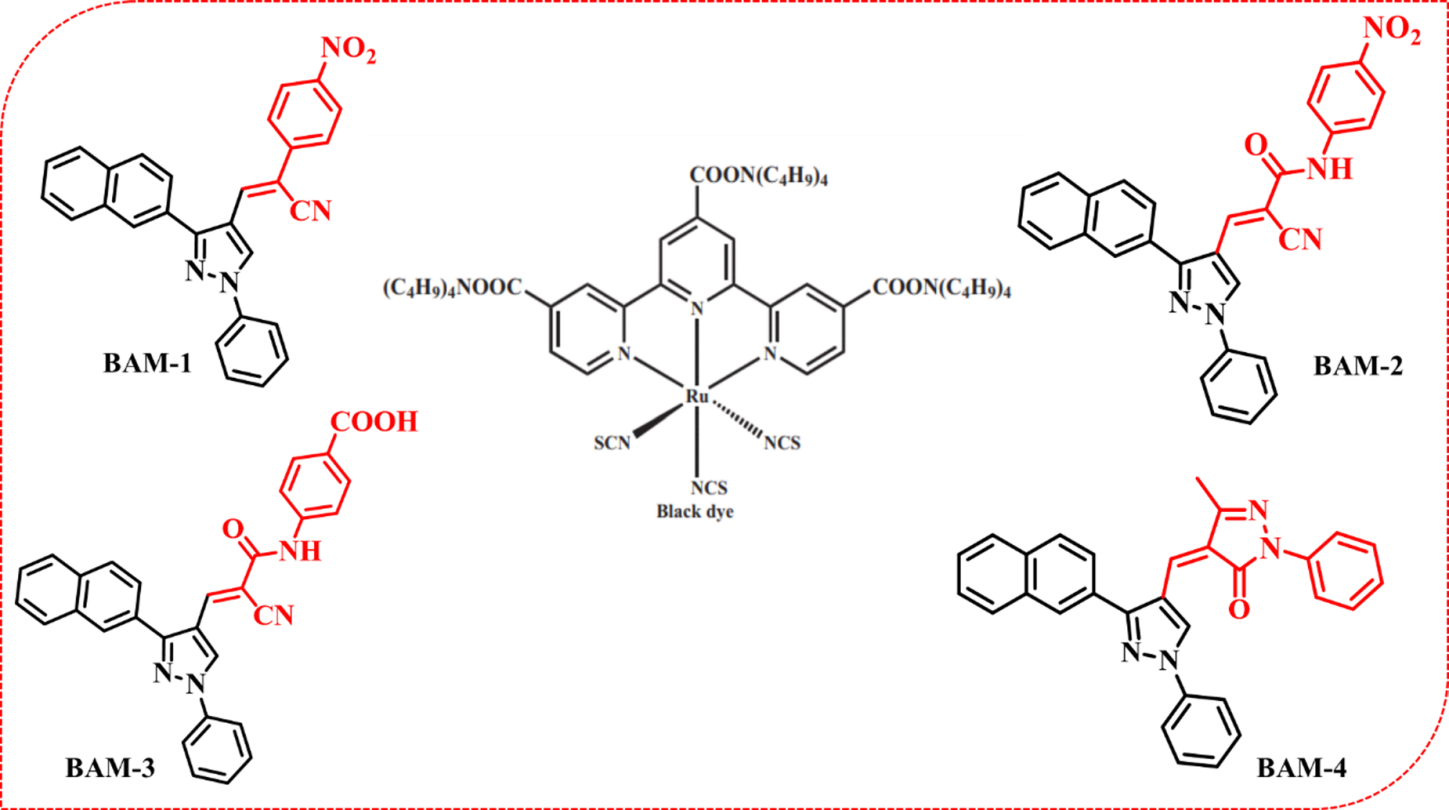
Molecular structures of naphthalene sensitizers **BAM-1–4.**

Dye-sensitized solar cells (DSSCs) have emerged as promising alternatives to traditional photovoltaic technologies due to their ease of fabrication, mechanical flexibility, cost-effectiveness, and relatively high efficiency under low-light conditions^1–3^. Since their introduction by O’Regan and Grätzel in 1991^4^, significant progress has been made in optimizing the photoelectrochemical performance of DSSCs through the development of novel sensitizers. The role of the dye is pivotal, as it governs sunlight absorption, facilitates charge separation, and drives electron injection into the conduction band of the semiconductor, typically TiO_2_^5,6^. A major challenge in DSSC development lies in expanding the absorption range of sensitizers and increasing their molar extinction coefficients to enhance photon capture. Traditionally, Ru(II)-based polypyridyl complexes have set the standard for high-efficiency DSSCs due to their excellent photostability and favorable electronic properties, achieving efficiencies above 10% under AM 1.5G illumination such ‘black dye’ (N749)^7,8^. However, the high cost, scarcity, and purification challenges of noble metal complexes have spurred the search for sustainable, metal-free organic alternatives^9^. Organic dyes offer several advantages: they are synthetically versatile, environmentally benign, and allow for rational molecular design to tune light-harvesting and energy alignment characteristics^10,11^. Over the past three decades, dye-sensitized solar cells (DSSCs) have undergone significant evolution through the synergistic advancement of sensitizers, electrolytes, and device architectures. Classical ruthenium-based dyes such as **N3, N719**, and N749 established the early benchmarks due to their strong metal-to-ligand charge-transfer (MLCT) absorption, robust anchoring on TiO_2_, and good stability; recent developments have further enhanced their performance using cobalt and copper redox shuttles and spacer-free architectures to achieve excellent indoor efficiencies. Metal-free organic dyes with donor–π–acceptor (D–π–A) architectures have emerged as the most promising class of sensitizers, enabling tunable absorption, high molar extinction coefficients, and versatile molecular engineering through modification of donor, π-bridge, and auxiliary acceptor units. According to recent design-rule studies, careful optimization of molecular planarity, conjugation length, and steric protection is crucial to suppress aggregation and enhance intramolecular charge transfer^12,13^. Porphyrin-based sensitizers, benefiting from strong Soret and Q-band absorption and high photovoltage when coupled with cobalt or copper electrolytes, have achieved power-conversion efficiencies exceeding 14% under AM1.5G and over 30% under indoor illumination through co-sensitization with complementary organic dyes^12,13^. Recent comprehensive reviews highlight that further progress depends on balancing light harvesting, charge transfer, and interfacial recombination across all components. Building on these insights, this work explores a co-sensitized system combining D–π–A-type BAM dyes with Black dye to achieve complementary spectral coverage, efficient charge transport, and improved device stability. Most high-performing organic dyes employ a donor–π–acceptor (D–π–A) framework, where a π-conjugated bridge connects an electron-donating unit to an electron-accepting anchor. This architecture promotes intramolecular charge transfer (ICT), stabilizes excited states, and ensures efficient charge injection at the dye–TiO_2_ interface^12^. By carefully engineering the donor, π-bridge, and acceptor units, researchers have achieved improved solar spectrum coverage, minimized recombination losses, and enhanced device stability^13^. In recent years, tandem dye-sensitized solar cells** (tandem DSSCs)** have gained attention as a strategy to further boost overall power conversion efficiency. These architectures typically combine two sensitizers with complementary absorption spectra in a parallel or series configuration, enabling better photon utilization across the visible spectrum^14,15^. Co-sensitization of dyes with complementary absorption profiles is a proven route to enhance J_SC_ by broadening spectral harvesting and mitigating competitive absorption/aggregation effects. State-of-the-art examples include organic porphyrin co-sensitization achieving > 14% under AM1.5G, parallel/tandem DSSCs with boosted current density, and molecular-interface engineering (e.g., hydroxamic-acid pre-adsorption) that optimizes co-adsorbed dye packing and further elevates device efficiency^15^. Such devices not only improve efficiency but are also well-suited for indoor photovoltaic applications, where spectral matching is critical. Motivated by these advances, we report a new class of naphthalene-based organic sensitizers (**BAM-1 to BAM-4**) designed within the D–π–A framework. These dyes incorporate a phenyl-pyrazole π-bridge and structurally diverse acceptor groups, including 4-nitroacetonitrile, 4-nitroacetamide, 4-carboxyacetamide, and pyrazalone. Naphthalene serves as a strong electron-donating unit due to its planarity and extended π-system, while the variation in acceptors allows fine-tuning of photophysical and electrochemical properties. We investigate the structure–property relationships of these dyes through UV–Vis spectroscopy, cyclic voltammetry, DFT calculations, and DSSC performance testing. Most efficient organic sensitizers adopt a (D–π–A) architecture, where a π-bridge mediates intramolecular charge transfer from an electron-rich donor to an electron-deficient anchoring acceptor. This spatially separated HOMO/LUMO distribution enables efficient electron injection into TiO_2_ and dye regeneration. Our **BAM-**series dyes follow this paradigm, employing a naphthalene donor and phenyl-pyrazole π-bridge coupled with acceptors of progressively varied electron-withdrawing strength (NO_2_ > CONHNO_2_ > CONHCOOH > pyrazolone), thereby tuning energy alignment and ICT efficiency. Such structural modulation of donor/π/acceptor units is well-known to govern absorption, energy levels, and PCE in D–π–A dyes Our findings reveal that **BAM-3** and** BAM-4**, when co-sensitized with **Black Dye** (Fig. 1) in a parallel tandem DSSC configuration, exhibit superior light absorption, improved interfacial charge transfer, and enhanced PCEs of 12.13% under simulated sunlight and over 25% under 1000 lx indoor illumination. These results demonstrate the potential of rational dye design and tandem integration for next-generation DSSCs optimized for both solar and indoor energy harvesting.

## Experimental section

### General synthesis of sensitizers (BAM-1)

In a 150 mL round-bottom flask, A combination of 3-(naphthalen-2-yl)-1-phenyl-1*H*-pyrazole-4-carbaldehyde (0.2 g, 0.006 mmol) **(1),** 2-(4-nitrophenyl)acetonitrile (0.11 g, 0.006 mmol) **(2),** 2-cyano-N-(4-nitrophenyl)acetamide (0.137 g, 0.006 mmol) **(3)**, 4-(2-cyanoacetamido)benzoic acid **(4)** and 5-methyl-2-phenyl-2,4-dihydro-3*H*-pyrazol-3-one (0.12 g, 0.006 mmol) **(5)** were refluxed in absolute ethanol (25 ml) with a drops of piperidine for 2 h. The progress of the reaction was assessed using thin-layer chromatography (TLC). The solid sensitizers **BAM-1–4** produced upon reflux and then filtered and dried.

### 3-(3-(Naphthalen-2-yl)-1-phenyl-1H-pyrazol-4-yl)-2-(4-nitrophenyl) acrylonitrile (BAM-1)

Yellow powder (95%), (m.p. 224–226 °C). IR (KBr): *ν*_max_ 2218 cm^−1^ (CN) and 1628 cm^−1^ (C=C). ^1^H NMR in DMSO-*d*_6_ *δ* 7.46 (t, *J* = 7.5 Hz, 1H, Ar–H), 7.57 (d, *J* = 7.5 Hz, 1H, Ar–H), 7.59 (d,* J* = 7.0 Hz, 1H, Ar–H), 7.60 (d, *J* = 7.5 Hz, 1H, Ar–H), 7.62 (d, *J* = 7.00 Hz, 1H, Ar–H), 7.88–7.90 (dd,* J* = 2.5, 9.00 Hz, 1H, Ar–H), 7.98 (d, *J* = 7.00 Hz, 2H, Ar–H), 8.00 (s, 1H, Ar–H), 8.00 (d,* J* = 7.5 Hz, 1H, Ar–H), 8.06 − 8.09 (m, 2H, Ar–H), 8.11 (s, 1H, Ar–H), 8.25 (s, 1H, Ar–H), 8.31 (s, 1H, CH–N), 8.33 (s, 1H, Ar–H), 9.28 ppm (s, 1H, C=CH). ^13^C NMR in DMSO-*d*_6_ *δ* 107.88, 116.09, 117.75, 119.44(2C), 124.54(2C), 126.21, 126.73(2C), 126.79, 127.01, 127.71, 127.83, 128.18, 128.57, 128.65, 128.83, 128.89, 129.98(2C), 132.95, 133.03, 137.02, 138.83, 139.82, 147.16, 153.70 ppm. The calculated analysis for C_28_H_18_N_4_O_2_ (442.48) yielded: C, 76.01; H, 4.10; N, 12.66%. experimental were found to be: C, 76.12; H, 4.14; N, 12.71%.

### 2-Cyano-3-(3-(naphthalen-2-yl)-1-phenyl-1H-pyrazol-4-yl)-N-(4-nitrophenyl) acrylamide (BAM-2)

Yellow crystals (89%), (m.p. above 300 °C). IR (KBr): *ν*_max_ 3367 cm^−1^ (N–H), 2208 cm^−1^ (CN), 1688 cm^−1^ (C=O), 1614 cm^−1^ (C=C). ^1^H NMR in DMSO-*d*_6_ *δ* 7.48 (t, *J* = 7.5 Hz, 1H, Ar–H), 7.57–7.64 (m, 4H, Ar–H), 7.87–7.92 (m, 3H, Ar–H), 7.98–8.02 (m, 3H, Ar–H), 8.07–8.10 (m, 2H, Ar–H), 8.23 (d,* J* = 10.00 Hz, 2H, Ar–H), 8.30 (d, *J* = 9.00 Hz, 2H, Ar–H), 9.28 (s, 1H, C=CH), 10.86 ppm (s, 1H, NH). ^13^C NMR (DMSO-*d*_6_): *δ* 104.77, 119.39, 119.79 (2C), 120.44 (2C), 122.02, 124.88(2C) 126.35, 126.54, 126.94, 127.21, 127.78, 128.24, 128.33, 128.56, 128.63, 128.76, 129.70, 129.88, 130.04 (2C), 133.01, 133.07, 138.67, 142.94, 143.16, 144.70, 154.78, 161.19 ppm. The calculated elemental analysis for C_29_H_19_N_5_O_3_ (485.50) were C, 71.74; H, 3.94; N, 14.43. The experimental were found to be: C, 71.85; H, 3.88; N, 14.38; %.

### 4-(2-cyano-3-(3-(naphthalen-2-yl)-1-phenyl-1H-pyrazol-4-yl) acrylamido) benzoic acid (BAM-3)

Red crystals (85%), with a m.p. 236–238 °C. IR (KBr): *ν*_max_ 3336 cm^−1^ (N–H), 2205 cm^−1^ (CN), 1687 cm^−1^ (C=O), 1605 cm^−1^ (C = C). ^1^H NMR in DMSO-*d*_6_ *δ* 7.47 (t, *J* = 8.00 Hz, 1H, Ar–H), 7.58 (d, 1H, *J* = 8.00 Hz, Ar–H), 7.62 (t, *J* = 8.00 Hz, 3H, Ar–H), 7.76 (d, *J* = 9.00 Hz, 3H, Ar–H), 7.87–7.91 (m, 3H, Ar–H), 7.98–8.01 (m, 3H, Ar–H), 8.07- 8.10 (m, 2H, Ar–H and C=CH), 8.28 (s, 1H, Ar–H), 9.26 (s, 1H, Ar–H), 10.57 (s, 1H, NH), 12.78 (s, 1H, COOH). ^13^C NMR (DMSO-*d*_6_): *δ* 105.08, 115.04, 117.78, 119.72 (2C), 120.02(2C), 126.27, 126.33, 126.89, 127.14, 127.75, 128.14, 128.37, 128.55 (2C), 128.71, 129.54, 129.99 (2C), 130.31(2C), 132.98, 133.02, 142.43, 142.57, 154.65, 160.77, 166.99 ppm. The calculated elemental analysis for C_30_H_20_N_4_O_3_ (485.50): C, 74.37; H, 4.16; N, 11.56% and experimental were: C, 74.48; H, 4.22; N, 11.65%.

### 5-Methyl-4-((3-(naphthalen-2-yl)-1-phenyl-1H-pyrazol-4-yl) methylene)-2-phenyl-2,4-dihydro-3H-pyrazol-3-one (BAM4)

Orange powder (75%), with a m.p. 230–232 °C. IR (KBr): *ν*_max_ 3050 (CH aliphatic), 1676 (C=O) and 1592 cm^−1^ (C=C). ^1^H NMR in DMSO-*d*_6_ *δ* 2.27 (s, 3H, CH_3_), 7.21 (t, *J* = 8.00 Hz, 1H, Ar–H), 7.45 (t, *J* = 8.5 Hz, 2H, Ar–H), 7.49 (d,* J* = 7.00 Hz, 1H, Ar–H), 7.61- 7.65 (m, 4H, Ar–H), 7.70 (s, 1H, C=CH), 7.94 (d,* J* = 8.00 Hz, 1H, Ar–H), 7.97 (t, *J* = 8.00 Hz, 4H, Ar–H), 8.03–8.04 (m, 1H, Ar–H), 8.14 (t,* J* = 9.00 Hz, 2H, Ar–H), 8.37 (s, 1H, CH–N), 10.23 ppm (s, 1H, Ar–H). The calculated elemental analysis for C_30_H_22_N_4_O (454.53) were: C, 79.27; H, 4.88; N, 12.33% and experimental were: C, 79.36; H, 4.82; N, 12.37%.

## Results and discussion

### Synthesis and structural characterization

**Fig. 2:**
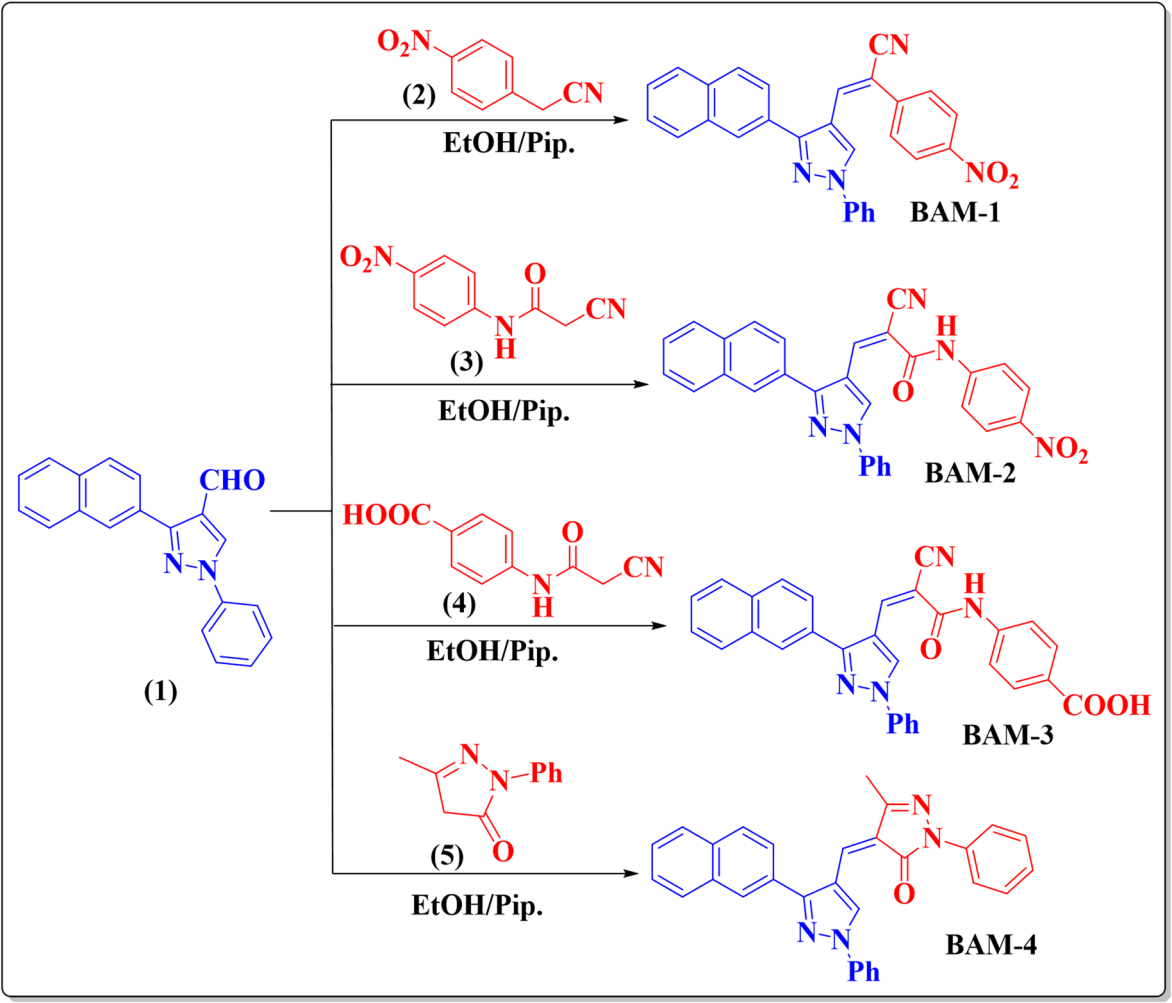
Synthesis of **naphthalene sensitizers BAM-1–4**.

The synthetic routes for the four novel naphthalene-based organic compounds **(BAM-1–4)** are shown in Fig. 2. Naphthalene sensitizers, specifically **BAM-1–4**, were synthesized in high yields via the Knoevenagel condensation of 3-(naphthalen-2-yl)-1-phenyl-1*H*-pyrazole-4-carbaldehyde (**1**) with 2-(4-nitrophenyl)acetonitrile (**2**), 2-cyano-N-(4-nitrophenyl)acetamide (**3**), 4-(2-cyanoacetamido)benzoic acid (**4**)^16^, and 5-methyl-2-phenyl-2,4-dihydro-3*H*-pyrazol-3-one (**5**), as depicted in Fig. 2. Spectral studies and elemental analyses validated the molecular structures of **BAM-1** to **BAM-4**. The IR spectra of **BAM-1** exhibit absorption bands attributed to the cyano group (CN) at 2218 cm^−1^. The ^1^H NMR spectra of **BAM-1** display singlet peak at *δ* 8.31 ppm for the (N–CH) proton. The singlet signal at *δ* 9.28 ppm is associated with the olefinic proton. The carbon from the cyano group (CN) appeared as a signal at *δ* 117.7 ppm in the ^13^C NMR spectra of **BAM-1**. The IR spectra of **BAM-2** exhibited vibrations that stretched bands at 3367 cm^−1^ and 2208 cm^−1^, associated with the N–H and CN groups, respectively, in addition to a prominent band at 1688 cm^−1^ attributable to the C=O group. The ^1^H NMR spectrum exhibited a singlet peak at *δ* 10.86 ppm, indicative of the (-NH) proton. Additionally, ^13^C NMR spectra of **BAM-2** showed a clear peak at 161.19 ppm, indicating the presence of a carbonyl group. The IR spectra of **BAM-3** exhibited stretching vibration bands at 3336 and 2205 cm^−1^, which indicate the N–H and C≡N groups, respectively, in conjunction with a prominent band at 1687 cm^−1^, ascribed to the C=O group. ^1^H NMR spectrum exhibited a multiplet signal at *δ* 8.07–8.10 ppm, indicative of aromatic and olefinic protons. The protons of the (N–H) and (COOH) groups displayed singlet signals at *δ* 10.57 and 12.78 ppm, respectively. The ^13^C NMR spectra of **BAM-3** displayed distinct signals at *δ* 117, 160.77, and 166.99 ppm, indicating the presence of cyano and two carbonyl groups. The methyl (CH_3_) group is indicated by a singlet signal at *δ* 2.27 ppm in the ^1^H NMR spectra of **BAM-4**. Within the range of *δ* 7.21–10.23 ppm, the aromatic protons exhibited triplet, doublet, multiplet, singlet, double, triplet, multiplet, triplet, and singlet signals. The vinylic proton and CH–N group protons produced singlet signals at *δ* 7.70 and 8.37 ppm, respectively. The spectral figures for the synthetic compounds are displayed in Figures S1–S11 (supplementary file).

### Photophysical properties of sensitizers BAM-1–4

**Fig. 3:**
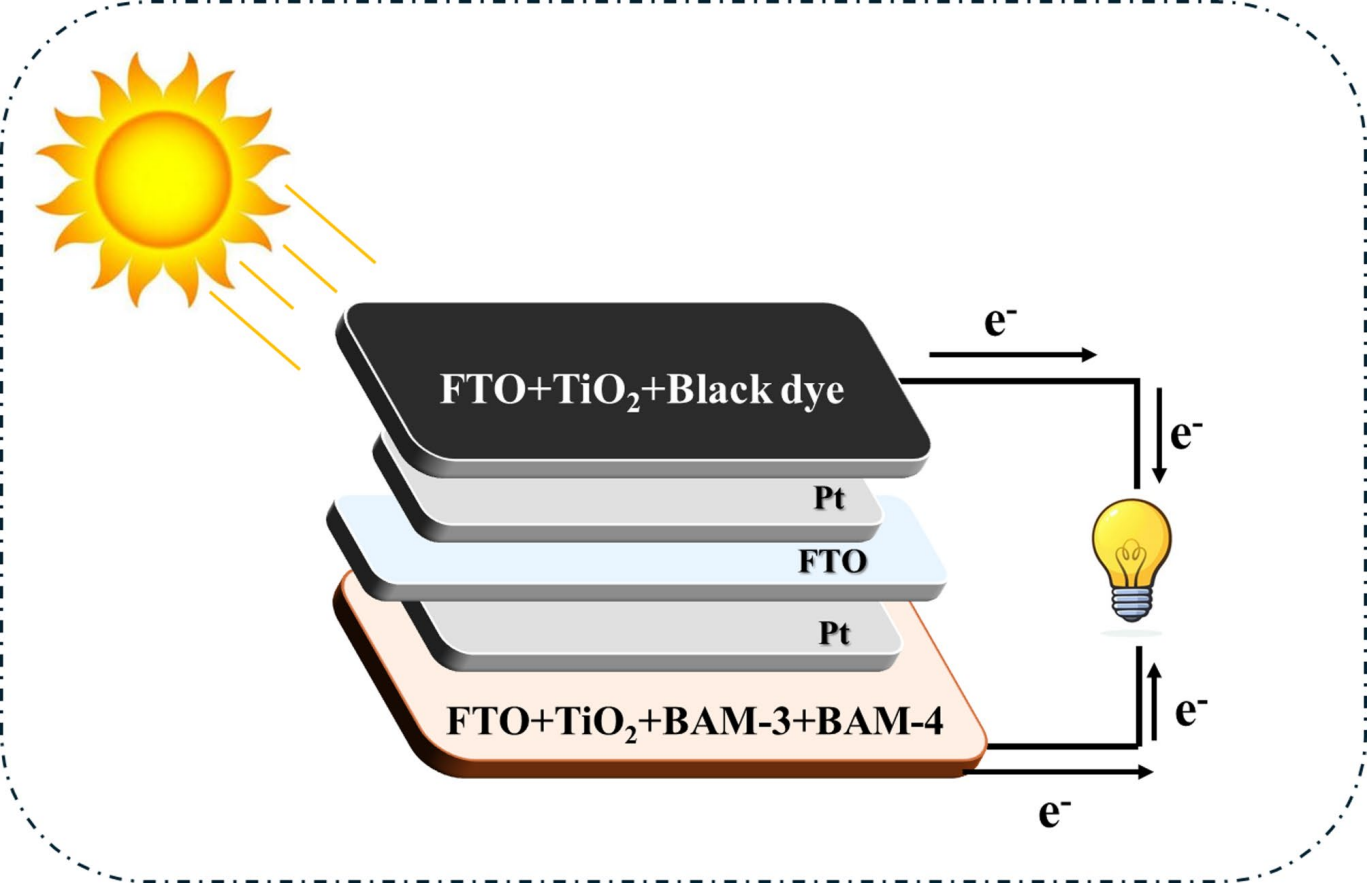
Structure of tandem devices by **BAM-3 + BAM-4** and **Black** sensitizers.

As depicted in Fig. 3, a parallel (PT-DSSC) was constructed utilizing **BAM-3, BAM-4,** and **Black Dye** as sensitizers. This tandem structure incorporates two photoanodes: the upper layer (**FTO/TiO**_**2**_**/Black Dye**) was optimized to capture light in the longer wavelength region, whereas the lower layer (**FTO/TiO**_**2**_**/BAM-3 + BAM-4**) effectively absorbed light at shorter wavelengths. The **PT-DSSC** was constructed using two photoanodes connected in parallel: the upper (**FTO/TiO**_**2/**_**Black Dye**) optimized for red-region absorption and the lower (**FTO/TiO**_**2**_**/BAM-3 + BAM-4**) for blue–green absorption. A dual-sided platinum foil served as both counter electrode and interlayer, enabling simultaneous operation of both sub-cells and efficient charge collection through parallel wiring. The electrolyte layer (I^−^/I_3_^−^) filled the interspaces between the electrodes, completing the tandem configuration. These two active layers are separated by a dual-sided platinum electrode that facilitates efficient charge transport and suppresses charge recombination. The strategic pairing of Black Dye with the **BAM-3** and **BAM-4** mixtures extended the overall light absorption range of the device, thereby improving the photogenerated charge density and enhancing the power conversion efficiency (PCE) of the system^17^.

**Fig. 4:**
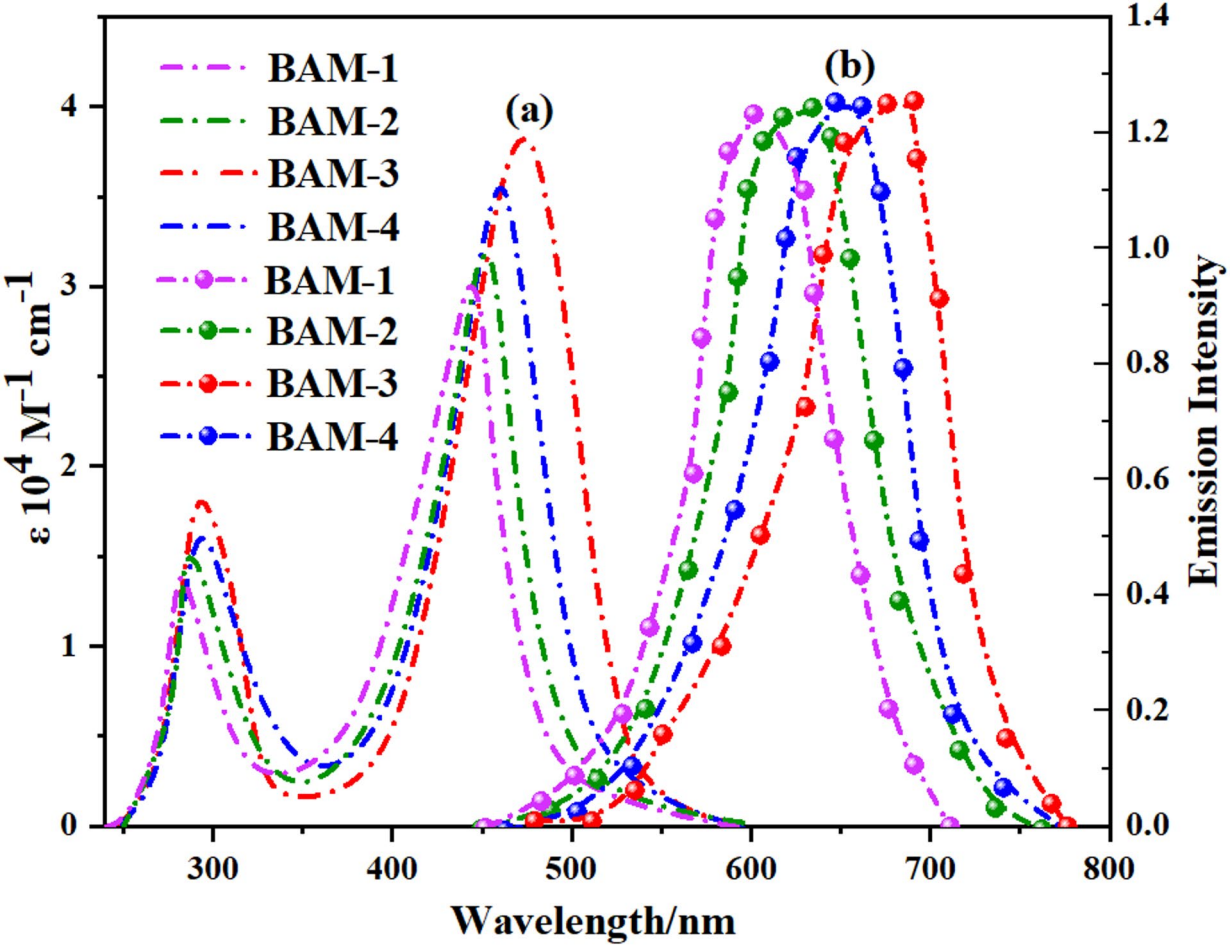
(**a**) UV–vis absorption and (**b**) emission spectra of naphthalene **BAM-1–4** sensitizers.

As shown in (Fig. 4a,b), the absorption and emission characteristics of **BAM-1 to BAM-4** sensitizers were thoroughly investigated using UV–visible spectroscopy, revealing two key absorption regions. From Table 1, The high-energy bands observed between 250 and 350 nm were assigned to localized π–π* transitions within the conjugated naphthalene framework. In contrast, broader absorption bands spanning 400–550 nm are indicative of intramolecular charge transfer (ICT) processes, in which electrons are transferred from the electron-rich naphthalene core to the electron-deficient end groups^18^.

**Table 1:** Photophysical properties of naphthalene sensitizers **BAM-1–4**.

Sensitizers	λ_max_^a^ (nm)	Ɛ (10^4^ M^−1^ cm^−1^)	λ_em_^b^ (nm)	Experimental E_0-0_^b^ (eV)
BAM-1	445	2.95	601	2.47
BAM -2	453	3.11	634	2.43
BAM-3	473	3.82	690	2.29
BAM-4	458	3.54	647	2.33

^a^Absorption and emission spectra were measured in DMF.

^b^E_0-0_ was determined from the intersection of the absorption and emission spectra in DMF.

The chemical nature of these acceptor units significantly influences electronic transitions^19^. **BAM-1** and **BAM-2**, incorporating 4-nitroacetonitrile and 4-nitroacetamide functionalities, respectively, displayed ICT maxima at 445 and 453 nm, along with moderate molar extinction coefficients (2.95 and 3.11 × 10^4^ M^−1^ cm^−1^) and relatively wide optical gaps of 2.47 and 2.43 eV. In comparison, **BAM-3**, which features a 4-carboxyacetamide group, exhibits a red-shifted λ_max_ at 473 nm, the highest molar absorptivity (3.82 × 10^4^ M^−1^ cm^−1^), and the narrowest band gap of 2.29 eV, indicating improved ICT efficiency and stronger absorption in the visible region. Similarly, **BAM-4**, which contains a pyrazolone acceptor, showed enhanced spectral coverage with λ_max_ at 458 nm, coupled with a high ε value of 3.54 × 10^4^ M^−1^ cm^−1^. These observations suggest that both **BAM-3** and **BAM-4** benefit from extended π-conjugation and better electronic coupling between the donor and acceptor moieties. The emission spectra (Fig. 4b) of the four sensitizers further corroborate their strong ICT nature, displaying broad fluorescence bands in the 600–700 nm region. The emission maxima (λ_em_) follow the order **BAM-3 (690 nm) > BAM-4 (647 nm) > BAM-2 (634 nm) > BAM-1 (601 nm)**. The trend in the energy gaps (E_0-0_), which decreases in order **BAM-1 > BAM-2 > BAM-4 > BAM-3**, supports this interpretation^20^. Notably, all four dyes demonstrated significantly stronger absorption than the reference ruthenium-based **black dye**. This highlights the improved light-harvesting capabilities of the** BAM** series. UV–Vis absorbation of compound BAM-1–4 and Black dye is displayed in Figure S12 (supplementary file).

**Fig. 5:**
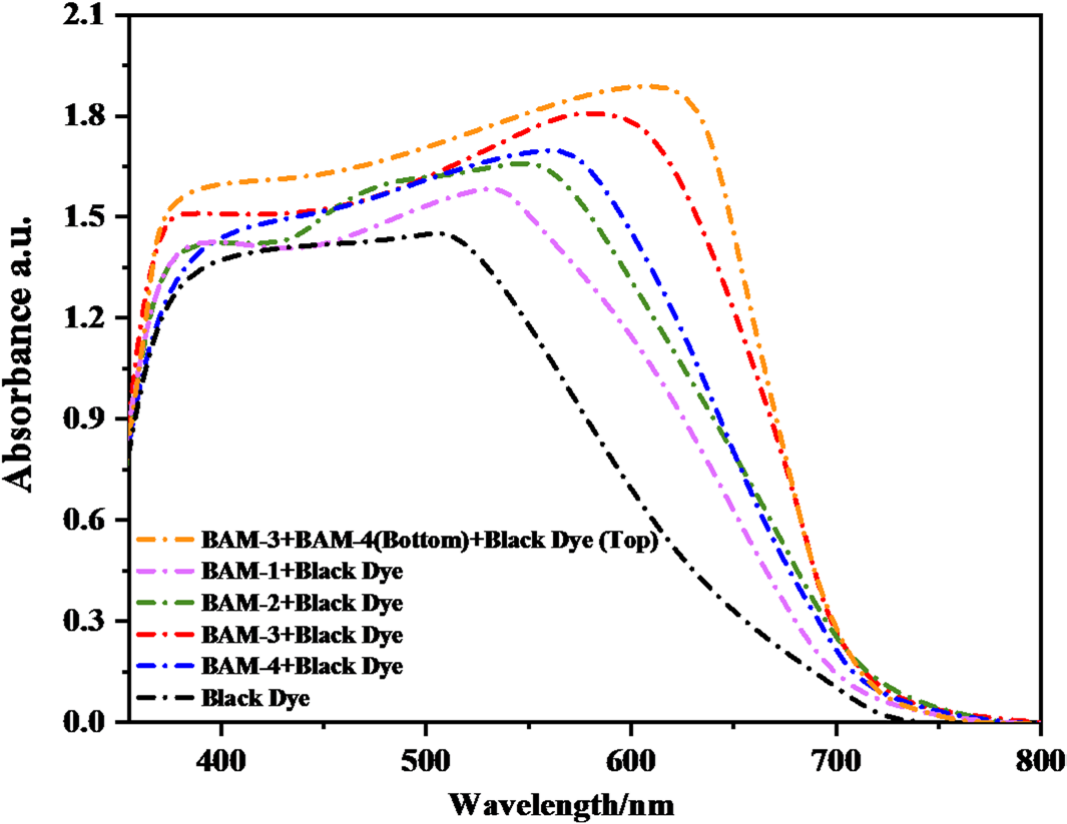
UV–visible spectra of **BAM-1–4 + Black Dye** and tandem devices by **BAM-3 + BAM-4** and **Black** sensitizers-anchored TiO_2_.

The UV–visible absorption spectra of **BAM-1** to **BAM-4** co-sensitized with Black Dye, along with the tandem-sensitized system combining **BAM-3, BAM-4**, and **Black Dye**, are shown in Fig. 5. All dye-sensitized TiO_2_ films demonstrate broad absorption spanning 300–50 nm, with enhanced spectral intensity and red-shifted features compared to individual dye spectra^21,22^. This enhancement is particularly notable for **BAM-3** and **BAM-4**, which possess stronger donor–acceptor conjugation and more delocalized electronic structures. Co-sensitization with Black Dye results in complementary light absorption, leading to improved coverage of the solar spectrum^23^. The tandem device, comprising **BAM-3** and **BAM-4** in combination with Black Dye, exhibits the most pronounced spectral broadening and red-shift, particularly beyond 600 nm. These spectral changes suggest the formation of* J*-type aggregates on the TiO_2_ surface^24^, which are known to enhance light absorption by promoting intermolecular π–π stacking and dipole alignment. The relatively sharp absorption bands of **BAM-3** and **BAM-4**, with minimal red-shift or broadening upon adsorption on TiO_2_, indicate limited dye aggregation. This behavior arises from steric hindrance introduced by the phenyl-pyrazole bridge and the bulky acceptor substituents, which disrupt parallel stacking interactions and maintain effective monolayer dispersion. Similar structural anti-aggregation strategies have been reported to enhance spectral stability and device reproducibility^24^. **BAM-3** contains a carboxylic acid group that chemisorbs TiO_2_ in carboxylate mode (commonly bidentate/bridging). **BAM-4** features a pyrazolone acceptor; its enolizable 1,3-dicarbonyl motif enables chelation to Ti(IV), analogous to β-diketone/barbituric anchors used in DSSCs. By contrast, **BAM-1** (4-nitroacetonitrile) and **BAM-2** (4-nitroacetamide) do not possess a classical –COOH/–PO_3_H_2_/–B(OH)_2_ anchor; their adsorption is weaker (H-bonding/Lewis coordination) and thus yields lower dye loading and robustness. Co-sensitization was performed sequentially, allowing the strongly anchored dye to establish a stable monolayer prior to co-adsorption of the second dye^25^. Additionally, the red-shift and increased intensity indicate efficient orbital overlap at the dye–semiconductor interface, facilitating stronger electronic coupling and more effective charge injection upon photoexcitation. Such behavior supports the observed improvement in photocurrent generation and device efficiency in the tandem configuration^23,26^.

### Electrochemical properties of BAM-1–4 sensitizers

The electrochemical behavior of the **BAM-1** to **BAM-4** dyes was investigated via cyclic voltammetry (CV) curves as depicted in (Fig. S13) to assess their suitability for electron injection into TiO_2_ and regeneration by the I^−^/I_3_^−^ redox couple^27–30^. The ground-state oxidation potentials (GSOP), which correspond to the HOMO energy levels, were calculated from the onset oxidation potentials, according to Eq. (1).

1 $$GSOP \, = [E_{onest}^{oxd} + 4.7)$$

**Fig. 6:**
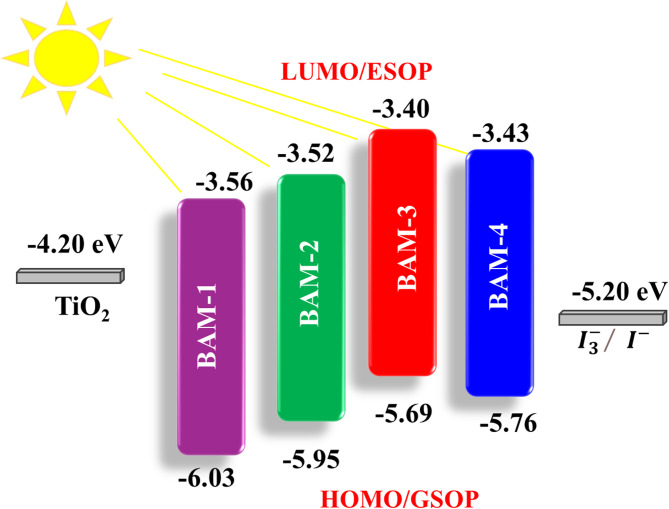
Experimental Energy levels of sensitizers **BAM-1–4.**

The excited-state oxidation potentials (ESOP), representing the lowest unoccupied molecular orbital (LUMO) levels, were subsequently calculated using Eq. (2):

2 $$ESOP \, = \, \left( {GSOP - \, E_{{0{-}0}} } \right)$$

where *E*_*0-0*_ is derived from the intersection of the absorption and emission. The measured HOMO levels for **BAM-1, BAM-2, BAM-3**, and **BAM-4** were − 6.03, − 5.95, − 5.69, and − 5.76 eV, respectively as shown in Fig. 6. These values indicate a sufficient thermodynamic driving force for dye regeneration by the I^−^/I_3_^−^ redox couple (− 5.20 eV), confirming the good oxidative stability of all the dyes. The corresponding LUMO levels (ESOPs) were calculated as − 3.56 eV for **BAM-1**, − 3.52 eV for **BAM-2,** − 3.40 eV for **BAM-3**, and − 3.43 eV for **BAM-4**, which lie well above the conduction band edge of TiO_2_ (− 4.20 eV), providing an ample energetic offset for efficient electron injection into the semiconductor as shown in Fig. 6. Among the series, **BAM-3** exhibited the narrowest band gap (2.29 eV) and the highest LUMO level (− 3.40 eV), suggesting the most favorable conditions for visible-light absorption and interfacial charge-transfer. These findings are consistent with the superior optical performance of **BAM-3** observed in previous **UV–Vis** studies. The gradual shift in both the HOMO and LUMO levels across the series reveals how the structural modifications of the acceptor groups fine-tune the electronic properties of the dyes. Overall, the energy level alignment of all four dyes supports their suitability for DSSC applications, with **BAM-3** offering optimal energetics for enhanced charge separation, electron injection, and dye regeneration efficiency.

### Molecular modeling of naphthalene BAM-1–4 sensitizers

**Fig. 7:**
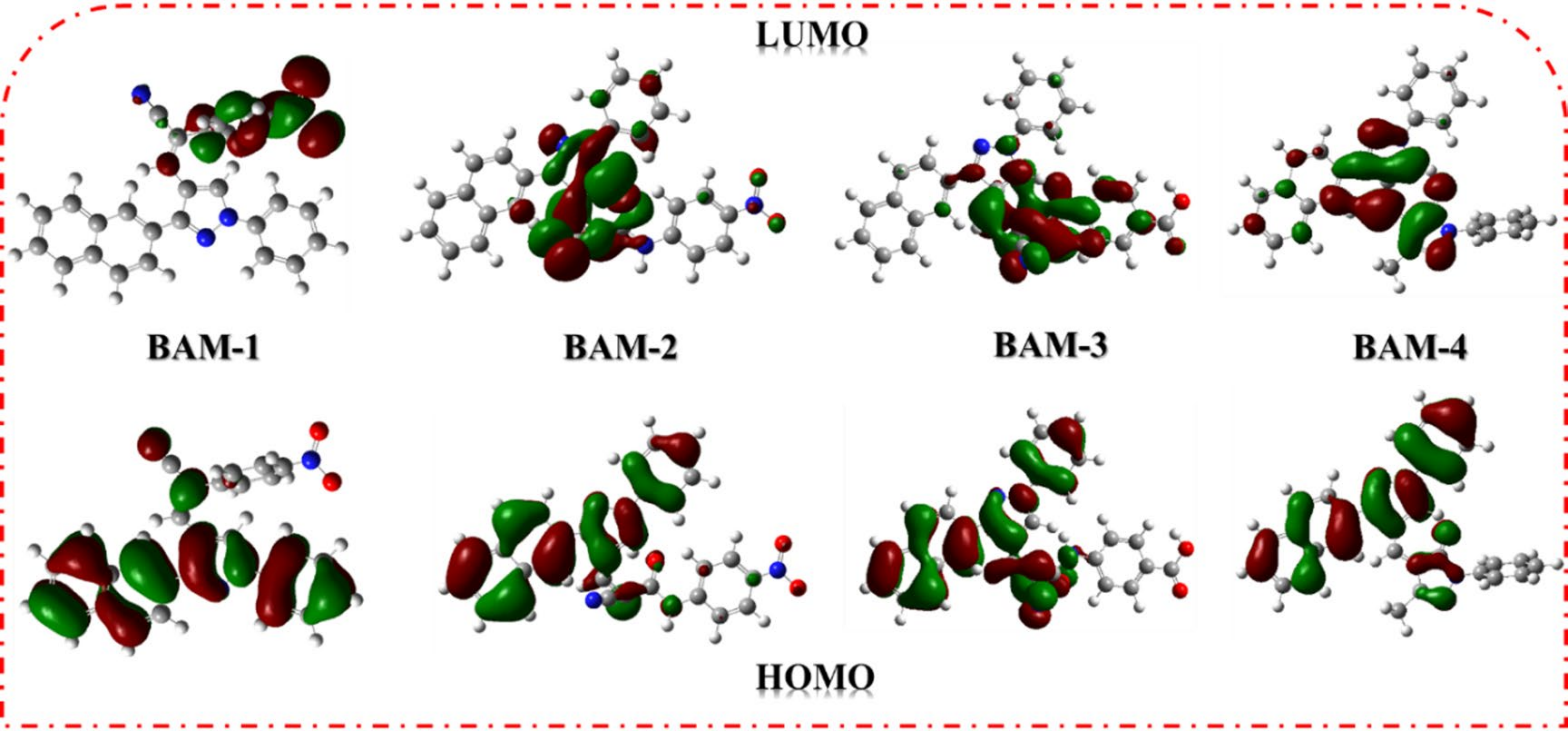
FMO in naphthalene** BAM-1–4** sensitizers.

Density Functional Theory (DFT) calculations were conducted on dyes **BAM-1–4** using the B3LYP/3-21G functional, implemented in the Gaussian09 software package^31^. The energy level graphs in Fig. 7. demonstrated the favorable alignment of the LUMO levels of the sensitizers relative to the TiO_2_ conductive band, which is essential for effective electron injection. The left naphthalene and phenyl-pyrazole moieties, together with the double bond, were the principal donors of the HOMOs in all compounds. Conversely, their LUMOs were mostly concentrated on the right-hand side, namely, on the anchoring groups^32^. For **the BAM-1** sensitizer dyes, the electron density of the HOMO was mostly localized on the donor components, whereas the electron density of the LUMO was primarily situated on the acceptor moieties of 4-nitroacetonitrile. This arrangement indicates inadequate electron transport from the HOMO to the LUMO levels, resulting in diminished electron efficiency. The incorporation of 4-nitrocyanoacetamide and carboxylic 4-cyanoacetamide into **BAM-2–3** altered the electron density distribution. For **BAM-2,** The electron density of the HOMO was primarily localized on the naphthalene, pyrazole, and phenyl donor components, whereas the electron density of the LUMO transitioned towards the acceptor groups of 4-nitroacetamide, as well as for **BAM-4** towards the acceptor groups (CN, CO, and COOH) of 4-carboxycyanoacetamide. Also, in case **BAM-4,** weakly charge transfer across acceptor moiety reflected on the process of (ICT). The calculated FMO distributions clearly indicate that the HOMOs are localized on the naphthalene and phenyl-pyrazole donor–π segments, whereas the LUMOs reside predominantly on the electron-accepting groups, confirming the D–π–A nature of **BAM-1** to **BAM-4**. The gradual increase in **HOMO–LUMO** delocalization from **BAM-1** to **BAM-4** reflects strengthened conjugation and ICT, in line with prior D–π–A systems that correlate structural extension with enhanced absorption and J_SC_.

### Molecular electrostatic potentials (MEP) of naphthalene sensitizers BAM-1–4

**Fig. 8:**
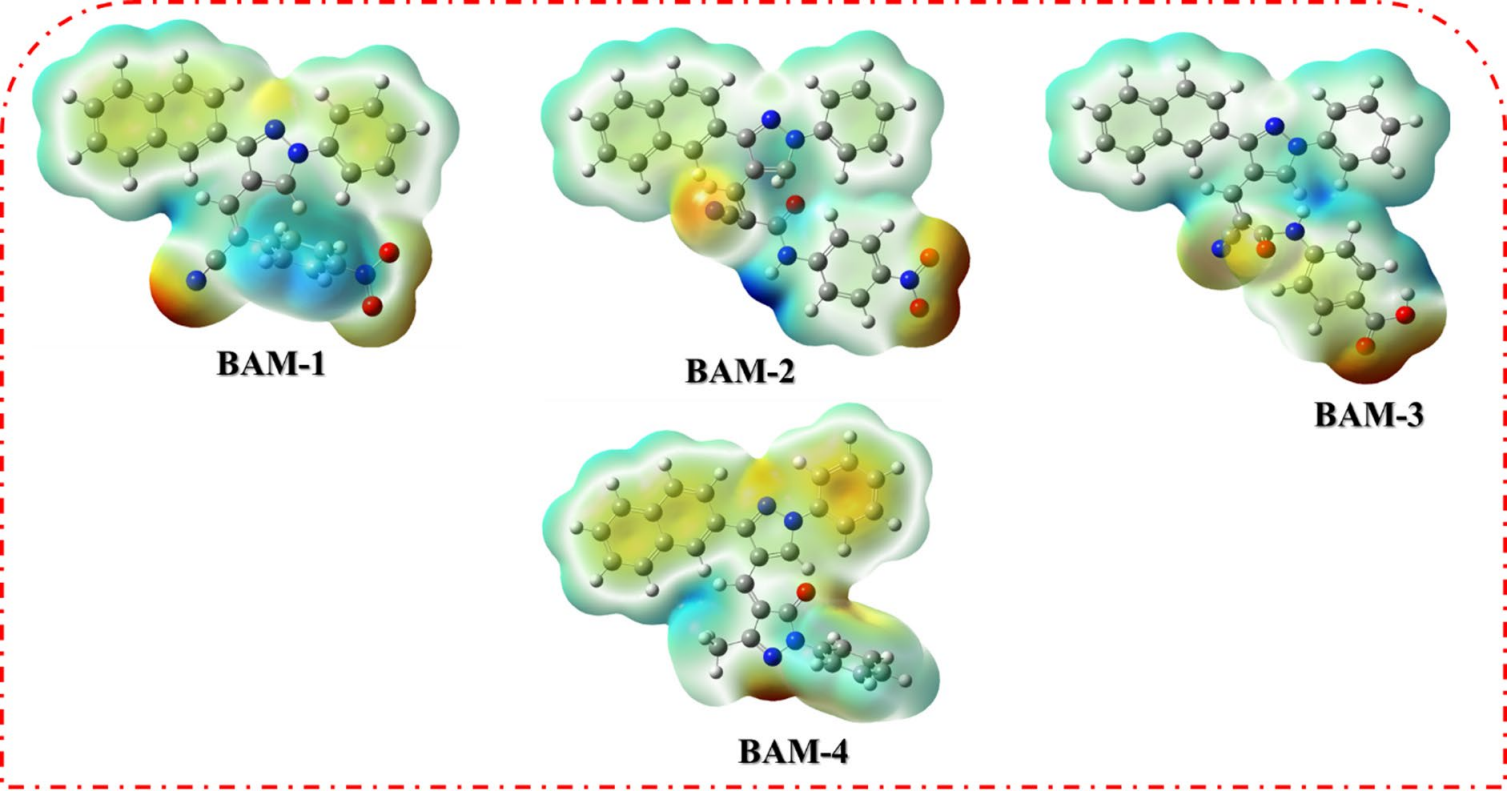
(MEP) of sensitizers **BAM-1–4.**

Molecular Electrostatic Potential (MEP) mapping provides valuable insights into the spatial distribution of electronic charges within dye molecules, which is critical for understanding their (ICT) behavior and electron-donating/accepting capabilities in (DSSCs)^33^. The MEP surfaces of **BAM-1** to **BAM-4** are presented in Fig. 8, where red and blue color gradients denote regions of negative and positive electrostatic potential, respectively. These maps help visualize electrophilic and nucleophilic reactive sites, offering a deeper understanding of charge delocalization across the dye frameworks. In the case of **BAM-1**, which incorporates a nitroacetonitrile acceptor group, the most intense negative potential is localized around the nitro (–NO_2_) and cyano (–CN) functionalities. This confirms the strong electron-withdrawing character of the acceptor unit, which plays a key role in stabilizing the LUMO and facilitating effective electron transfer from the donor. The donor region, comprising the naphthalene unit, exhibited a concentrated area of positive potential, validating its function as an electron source during photoexcitation. For **BAM-2**, which features a 4-nitroacetamide acceptor, the negative electrostatic potential is primarily associated with the nitro and amide oxygen atoms, indicating a strong electron affinity and localized charge trapping. Meanwhile, the donor core continued to display positive potential, suggesting a consistent D–π–A character and favorable ICT behavior. The electron density distribution is slightly more delocalized than that of **BAM-1**, indicating an improvement in intramolecular charge mobility. In **BAM-3**, which bears a carboxyacetamide group, the MEP map reveals a more uniform distribution of negative potential across the carboxyl and amide moieties. This suggests better conjugation between the acceptor and π-bridge, facilitating smoother electron delocalization upon excitation. The donor domain, again located on the naphthalene unit, maintained a strong positive potential, which supported the directional charge flow and efficient separation of excited-state charges. **BAM-4**, which contains a pyrazolone acceptor ring, exhibited a distinctly broadened negative electrostatic region encompassing both the pyrazolone core and adjacent electronegative atoms. This indicates an extended electron-withdrawing field that stabilizes the excited state and enhances the ICT. The progressive delocalization of the negative electrostatic potential from **BAM-1** to **BAM-4** reflects the strengthening of the acceptor strength and improved charge-separation efficiency.

### Electron localization function (ELF) of BAM-1–4

**Fig. 9:**
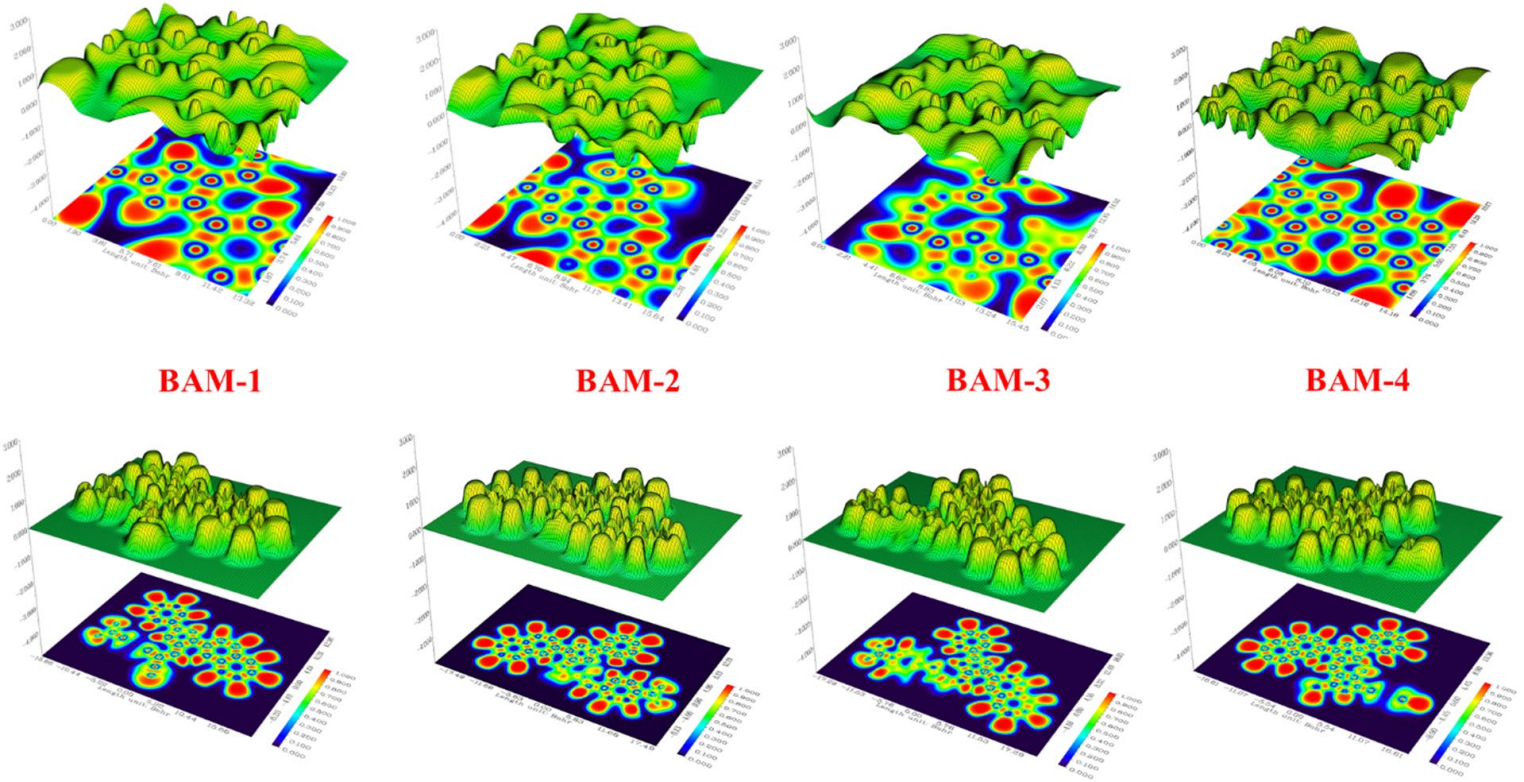
ELF maps of sensitizer **BAM-1–4**.

(ELF) surfaces of **BAM-1** to **BAM-4** (Fig. 9) provide valuable insights into the spatial distribution of electron density and the extent of intramolecular conjugation across the dye molecules^34^. In **BAM-1**, the ELF surface reveals sharp and well-defined peaks concentrated around the donor and π-bridge regions, indicating a highly localized electron density and limited delocalization. This constrained distribution may negatively affect the efficiency of (ICT). **BAM-2** exhibited a moderately smoother ELF profile, with partial delocalization extending toward the acceptor unit. **BAM-3** exhibited a more continuous and interconnected ELF surface, with a broader electron density across the donor-π-acceptor regions. This reflects enhanced π-conjugation and more efficient electron delocalization, which supports improved donor–acceptor interactions and charge transport. Among all the **BAMs**, **BAM-4** exhibited the most uniform and extensive ELF distribution. The smooth surface topology and widespread electron density are indicative of effective electronic coupling along the molecular backbone. This delocalization facilitates a highly efficient ICT process, which is essential for optimal performance in DSSC.

## Photovoltaic device characterization of BAM-1–4 sensitizers

**Fig. 10:**
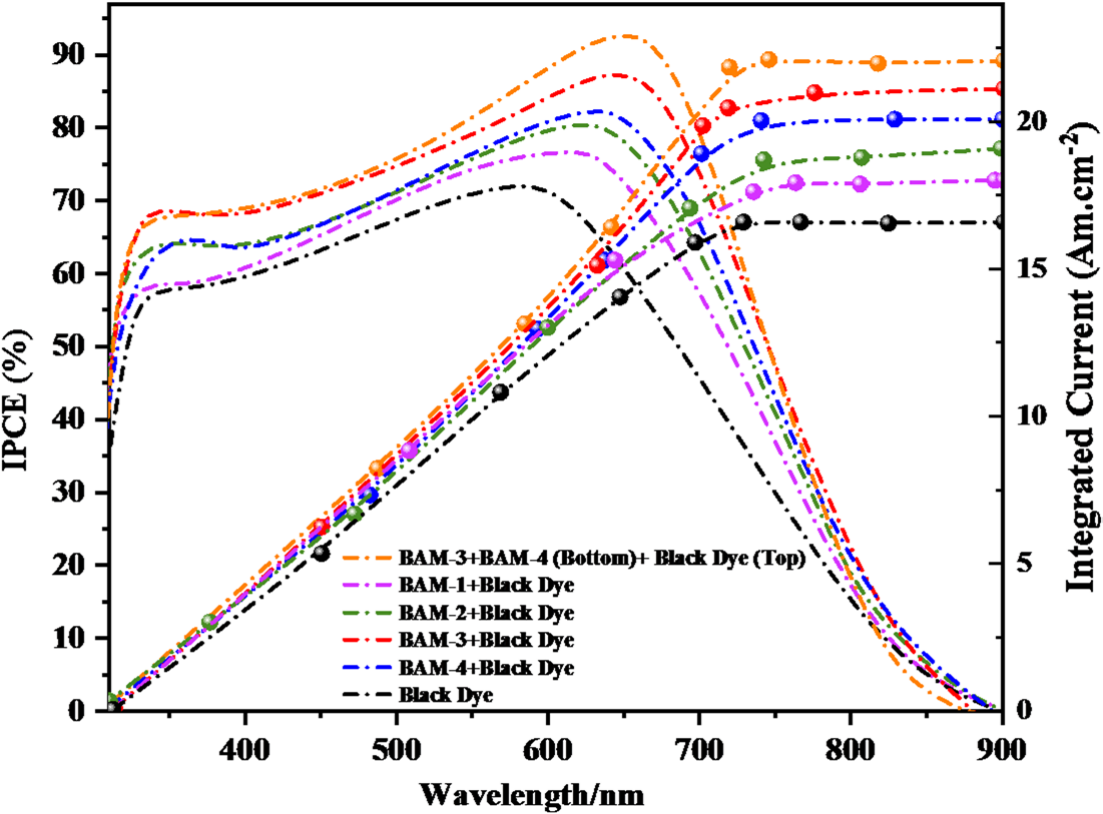
*IPCE* spectra of **BAM-1–4 + Black Dye** and tandem devices by **BAM-3 + BAM-4** and **Black Dye.**

The incident photon-to-current efficiency (*IPCE*) spectra of (DSSCs) fabricated using **BAM-1** to **BAM-4** in combination with **Black Dye**, along with a mixed (PT-DSSC) employing **BAM-3 + BAM-4** and **Black Dye**, are shown in Fig. 10. All devices exhibit broad photo-responses in the visible region (300–700 nm), with significant differences in peak intensity and spectral coverage reflecting the molecular design of the organic sensitizers and their synergy with Black Dye. Among the single co-sensitized devices, the **BAM-3 + Black Dye** system delivered the highest *IPCE*, reaching a peak of approximately 85.5%. This enhanced response is attributed to the structural characteristics of **BAM-3**, which features a 4-carboxyacetamide acceptor. This group enables strong electronic coupling with TiO_2_, promoting efficient (ICT), high dye loading, and effective electron injection. **BAM-4**, which contains a pyrazolone-based acceptor, also exhibited high *IPCE* performance (≈ 82%), attributed to its extended π-conjugation and compact five-membered ring structure, which facilitates broad absorption and favorable orbital alignment with the semiconductor^35^. In contrast, **BAM-1** and **BAM-2**, bearing nitroacetonitrile and 4-nitroacetamide acceptors respectively, showed lower peak *IPCEs* (≈ 76–79%). These results suggest a less efficient ICT and a narrower light-harvesting range, consistent with their weaker electron-withdrawing capacity and less delocalized electronic structures. The highest* IPCE* across all samples was observed in the **PT-DSSC** incorporating** BAM-3 + BAM-4 in the bottom photoanode and Black Dye** in the top photoanode, which achieved a remarkable peak *IPCE* of approximately 92%. This improvement arises from the complementary absorption profiles of the three dyes: while Black Dye contributes strongly to the red and near-infrared region, **BAM-3** and **BAM-4** effectively capture light in the blue-green part of the spectrum. The parallel tandem configuration enables these sensitizers to operate simultaneously, extending the spectral response and improving charge generation without competitive light absorption or recombination losses^36,37^. Additionally, the optimized dye loading and surface coverage enhance the photon-to-electron conversion across the full solar spectrum. Compared to the *Jsc* values obtained from the *J-V* data, the *Jsc*^*IPCE*^ values integrated from the *IPCE* spectra are quite consistent.

**Fig. 11:**
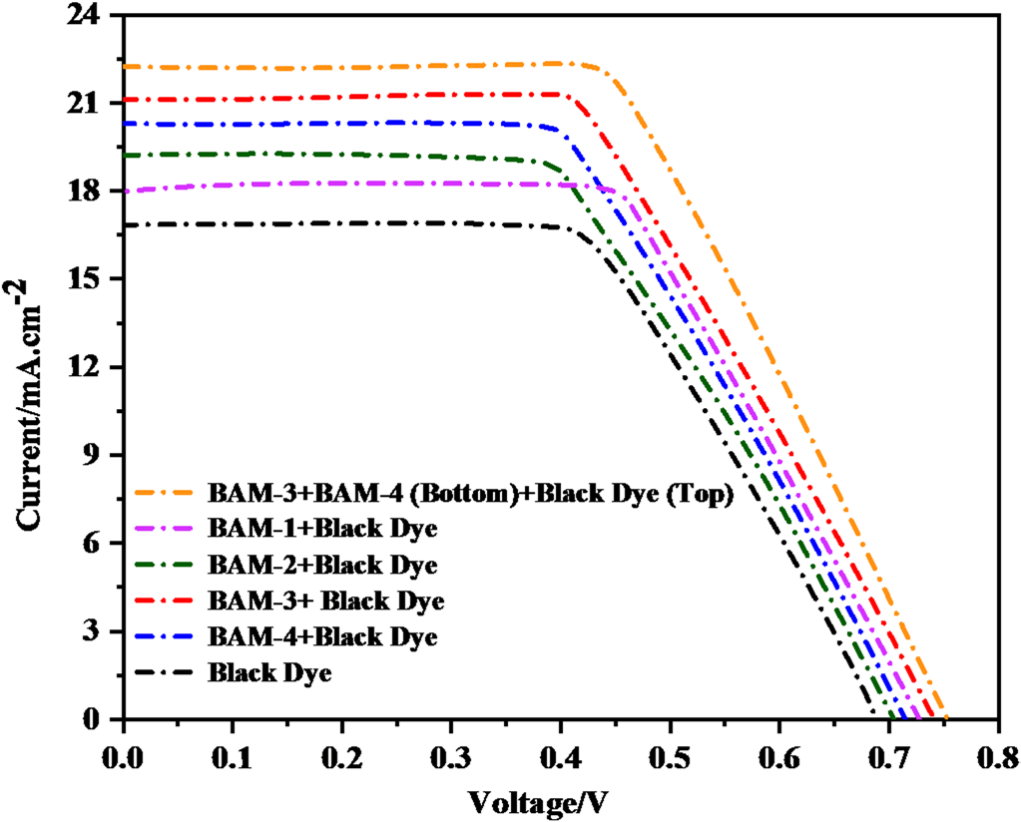
*J-V* plots of **BAM-1–4 + Black Dye** and tandem devices by **BAM-3 + BAM-4** and **Black Dye**.

As shown in Fig. 11, the photovoltaic parameters of DSSC devices based on **Black Dye**, individual combinations of **BAM-1** to **BAM-4** with Black Dye, and a parallel tandem architecture employing (**Black Dye (top) and (BAM-3 + BAM-4 (bottom)**) were systematically evaluated under standard AM 1.5G illumination. The corresponding values of *(V*_*OC*_), *(J*_*SC*_*),* fill factor *(FF), (η*_*cell*_*),* and dye-loading concentration are summarized in Table 2. **Black Dye** device displayed a *V*_*OC*_ of 0.665 V, *J*_*SC*_ of 16.68 mA/cm^2^, *FF* of 67.56%, and an overall efficiency of 7.49%. Notable improvements in both the photocurrent and device efficiency were observed upon introducing co-sensitization strategies with **BAM-1** through **BAM-4**, which were attributed to the enhanced spectral coverage and improved charge-transfer dynamics^37^. Among the co-sensitized systems, the (**BAM-3 + Black Dye)** device exhibited the best performance, achieving a *J*_*SC*_ of 21.15 mA/cm^2^ and *η*_*cell*_ of 9.85%. This enhancement is attributed to the strong electron-withdrawing 4-**carboxyacetamide** group in **BAM-3**, which supports efficient (ICT), strong anchoring to TiO_2_, and a high dye-loading capacity (3.82 × 10^–5^ mol/cm^2^). **BAM-4** also contributed significantly to device performance, delivering a* J*_*SC*_ of 20.38 mA/cm^2^ and *η*_*cell*_ of 8.89%, due to its pyrazolone acceptor group that promotes extended conjugation and spectral broadening. Devices based on **BAM-1** and **BAM-2** with **Black Dye** also showed respectable efficiencies of 7.95% and 8.26%, respectively, with slight variations in the *V*_*OC*_ and *FF* values, likely influenced by differences in the molecular structure and dye packing on the TiO_2_ surface. Under identical terminal voltage, the PT-DSSC current equals the sum of the branch currents from the top (Black dye) and bottom (BAM-3/4) photoanodes. The modest increase in Jsc relative to single devices arises because the bottom cell receives a spectrally filtered and partially attenuated photon flux after passing through the top TiO_2_/Black-dye layer and the dual-sided Pt interlayer. Additionally, moderate spectral overlap and thickness/dye-loading choices made to maintain fast transport constrain the incremental bottom-cell current. Similar *Jsc* behavior has been noted in optimized tandem DSSCs and hybrid tandems when interlayer transparency and current sharing are the primary bottlenecks^37^. The obtained photovoltaic performance exhibited a significant improvement compared to the previously reported literature data, as shown in Table S1 of the supplementary file. Additionally, the optimized, CDCA-free dye adsorption conditions used for device fabrication are summarized in Table S2 of the supplementary file.

**Table 2:** Photovoltaic values of **BAM-1–4 + Black Dye** and tandem devices by **BAM-3 + BAM-4** and **Black Dye.**

Sensitizer	*Device type*	*V*_*OC*_ * (eV)*	*J*_*SC*_ (mA/cm^2^)	*FF %*	η_cell_ (%)	Concentration of the dye/10 ^−5^ mol cm^−2^
Black Dye	–	0.665	16.68	67.56	7.49	3.23
BAM-1 + Black Dye	–	0.726	18.05	60.67	7.95	3.30
BAM-2 + Black Dye	–	0.689	19.17	62.54	8.26	3.67
BAM-3 + Black Dye		0.737	21.15	63.23	9.85	3.82
BAM-4 + Black Dye		0.714	20.38	61.12	8.89	3.51
Black Dye (Top) + BAM-3 + BAM-4 (Bottom)	P-Tandem	0.754	22.19	72.49	12.13	3.93

The most significant advancement was achieved with the parallel tandem DSSC configuration, incorporating a Black Dye at the top photoanode and a **BAM-3 + BAM-4** blend at the bottom electrode. This architecture delivered a remarkable *V*_*OC*_ of 0.754 V, *J*_*SC*_ of 22.19 mA/cm^2^, *FF* of 72.49%, and an outstanding overall efficiency of **12.13%**, which is a ~ 70% enhancement compared to the **Black Dye-only** device. The parallel connection lowers the effective series resistance and raises the apparent shunt resistance, producing a sharper I–V curve and higher FF. In our stack, the dual-sided Pt interlayer reduces charge-transfer resistance and supports efficient mass transport, further enhancing FF; this interpretation is consistent with our EIS, which shows increased recombination resistance for the tandem device. Literature on tandem/dual-photoanode DSSCs reports similar FF improvements upon introducing low-resistance interfacial layers. The synergistic interaction between the complementary absorption profiles of **BAM-3** and **BAM-4** at the bottom and the **Black Dye** at the top resulted in more efficient solar spectrum utilization and enhanced photo-induced charge generation. Furthermore, the increased dye-loading concentration (3.93 × 10^–5^ mol/cm^2^) in the tandem system contributed to higher photon absorption and current output. These results clearly demonstrate that the structural tailoring of organic sensitizers, when coupled with tandem co-sensitization strategies, can significantly elevate the efficiency of DSSCs. In particular, the tandem configuration based on **BAM-3** and **BAM-4** in combination with **Black Dye** offers a promising path toward next-generation high-efficiency dye-sensitized photovoltaic devices^37,38^.

**Fig. 12:**
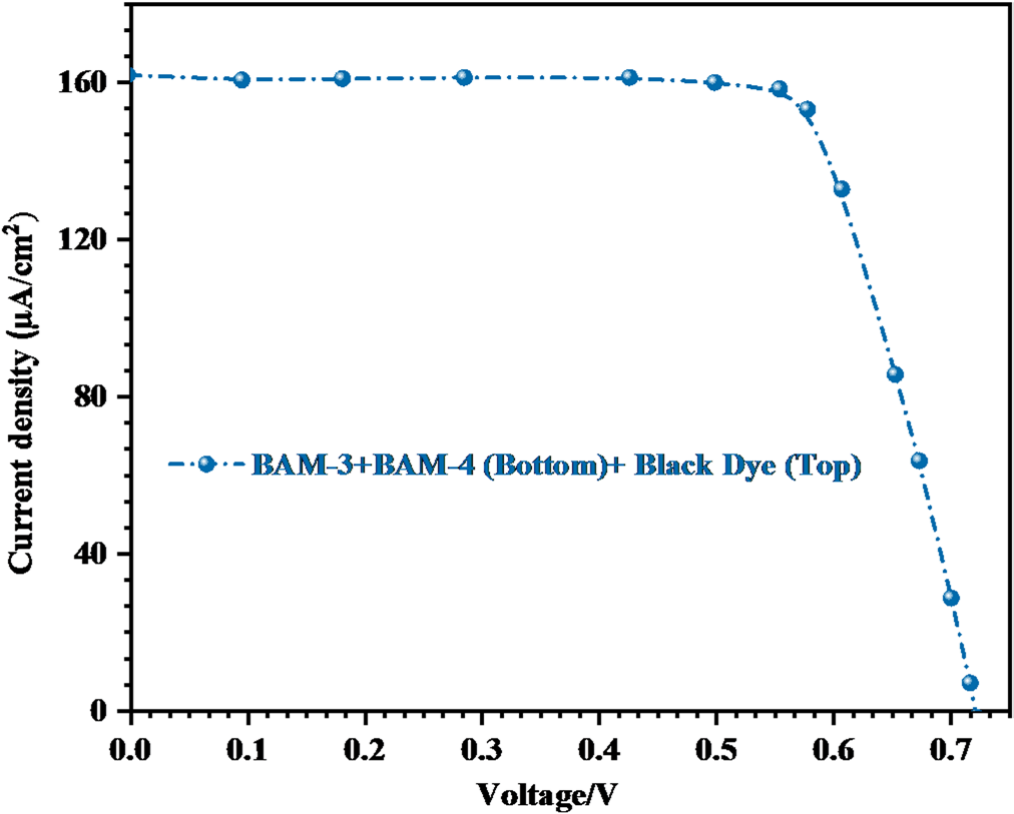
*J* − *V* curve for tandem devices by **BAM-3 + BAM-4** and **Black Dye** under 1000 lx illumination.

Under indoor illumination conditions, (PT-DSSC) incorporating **Black Dye** as the top absorber and **BAM-3 + BAM-4** as co-sensitizers in the bottom cell exhibited a remarkable (PCE) of 25.85%, as shown in Fig. 12 and summarized in Table 3.

**Table 3:** *J-V* parameters at 1000 lx (0.283 mW.cm^−2^) for tandem **(BAM-3 + BAM-4 (bottom) + Black Dye (top).**

Sensitizer (0.2 m)	*Device type*	*V*_*OC*_ * (eV)*	*J*_*SC*_ (μA/cm^2^)	*FF %*	*η*_*cell*_ * (%)*
Black Dye (Top) + BAM-3 + BAM-4 (Bottom)	P-Tandem	0.716	161.56	62.98	25.85

This outstanding performance is attributed to the synergistic interaction between the two dye components in the bottom cell, which offers complementary absorption spectra, and the strong visible-light response of the **Black Dye** in the top cell^39,40^. The tandem device achieved a *V*_*OC*_ of 0.716 V,* J*_*SC*_ of 161.56 μA cm^−2^, and* FF* of 62.98%. These values highlight the highly efficient charge separation and minimal recombination under low-light conditions. The enhanced photocurrent reflects the improved light harvesting from the broad spectral coverage enabled by the dual-dye strategy.

**Fig. 13:**
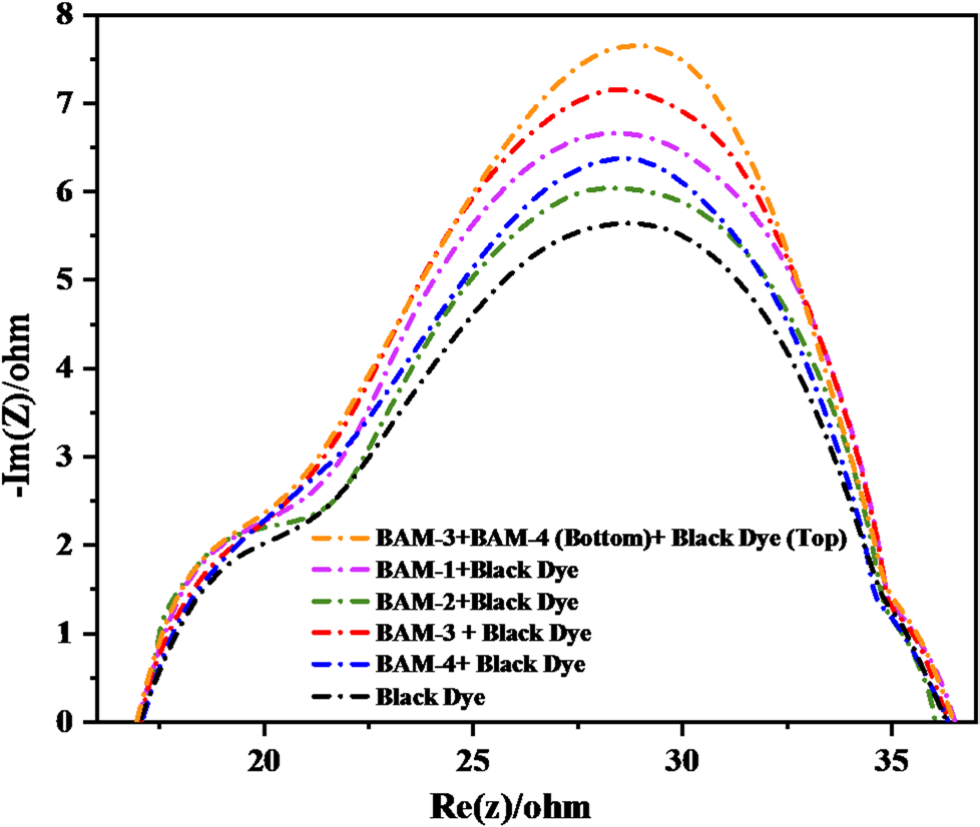
Nyquist curves of **BAM-1–4 + Black Dye** and tandem devices by **BAM-3 + BAM-4** and **Black Dye.**

To gain deeper insight into the interfacial charge transfer dynamics and recombination behavior in (DSSCs), electrochemical impedance spectroscopy (EIS) was performed under dark conditions at open-circuit voltage^11^. The Nyquist plots (Fig. 13) of the devices based on BAM-1 to BAM-4 co-sensitized with **Black Dye**, as well as the mixed PT-DSSC incorporating **BAM-3, BAM-4,** and **Black Dye**, reveal distinct semicircular features corresponding to the charge transport and recombination processes at the photoanode/electrolyte interface. All spectra display two characteristic semicircles: the high-frequency semicircle is attributed to the charge transfer resistance at the counter electrode *(R*_*ct*_*)* while the middle-to-low-frequency semicircle corresponds to the electron recombination resistance at the TiO_2_/dye/electrolyte interface *(R*_*rec*_*)*. The diameter of the second semicircle is particularly informative as it reflects the recombination resistance and thus the extent of the electron lifetime in the photoanode. Among the co-sensitized devices, **BAM-3 + Black Dye** and **BAM-4 + Black Dye** exhibited notably larger semicircle diameters than **BAM-1** and **BAM-2**, indicating higher recombination resistance and longer electron lifetimes. This suggests that **BAM-3** and **BAM-4** form more effective blocking layers on the TiO_2_ surface, possibly because of their stronger anchoring interactions or superior molecular packing, which help suppress back electron transfer to the electrolyte. The mixed **PT-DSSC**, which combined **BAM-3, BAM-4,** and **Black Dye**, showed the largest semicircle in the mid-frequency region, demonstrating the highest recombination resistance among all the tested configurations. This enhanced resistance is indicative of a significantly reduced recombination rate, which contributes directly to the elevated (*V*_*OC*_) and improved charge collection efficiency observed in this device^11^. The synergistic interplay between the dyes in the tandem configuration appears to facilitate more effective surface passivation and interfacial stability, thereby minimizing energy losses and improving overall photovoltaic performance.

(EIS) was used to further elucidate charge-transport and recombination behaviors in all devices. The extracted parameters, including charge-transport resistance (R_tr_), recombination resistance (R_rec_), electron lifetime (τ_e_), chemical capacitance (C_μ_), and charge-collection efficiency (η_cc_) are summarized in Table 4.

**Table 4:** Electrochemical parameters of **BAM-1–BAM-4 + Black Dye** and tandem devices (from EIS measurements at open-circuit voltage under dark conditions).

Device	R_tr_ (Ω)	R_rec_ (Ω)	τ_e_ (ms)	C_μ_ (μF)	η_cc_ (%)
Black Dye	22.8	59.4	23.1	41.2	61.6
BAM-1 + Black Dye	20.1	82.5	31.6	47.9	75.6
BAM-2 + Black Dye	18.7	95.2	36.4	52.5	80.3
BAM-3 + Black Dye	16.4	127.6	49.8	59.4	87.1
BAM-4 + Black Dye	17.5	119.3	46.7	57.6	85.3
PT-DSSC (BAM-3 + BAM-4 // Black Dye)	14.2	168.5	62.9	68.7	91.6

The **PT-DSSC** based on **BAM-3 + BAM-4** and **Black Dye** exhibited the highest *R*_*rec*_ and *τe,* indicating the most effective suppression of charge recombination and the longest electron lifetime. The *η*_*cc*_ exceeded 90%, confirming highly efficient charge collection at the TiO_2_/electrolyte interface. The smaller *R*_*tr*_ observed for the tandem configuration also reflects improved electron mobility, in agreement with its superior *FF* and *PCE*. These findings are consistent with earlier reports highlighting that enhancing *R*_*rec*_*/R*_*tr*_ ratios and *η*_*cc*_ are key to improving DSSC efficiency^11^.

**Fig. 14:**
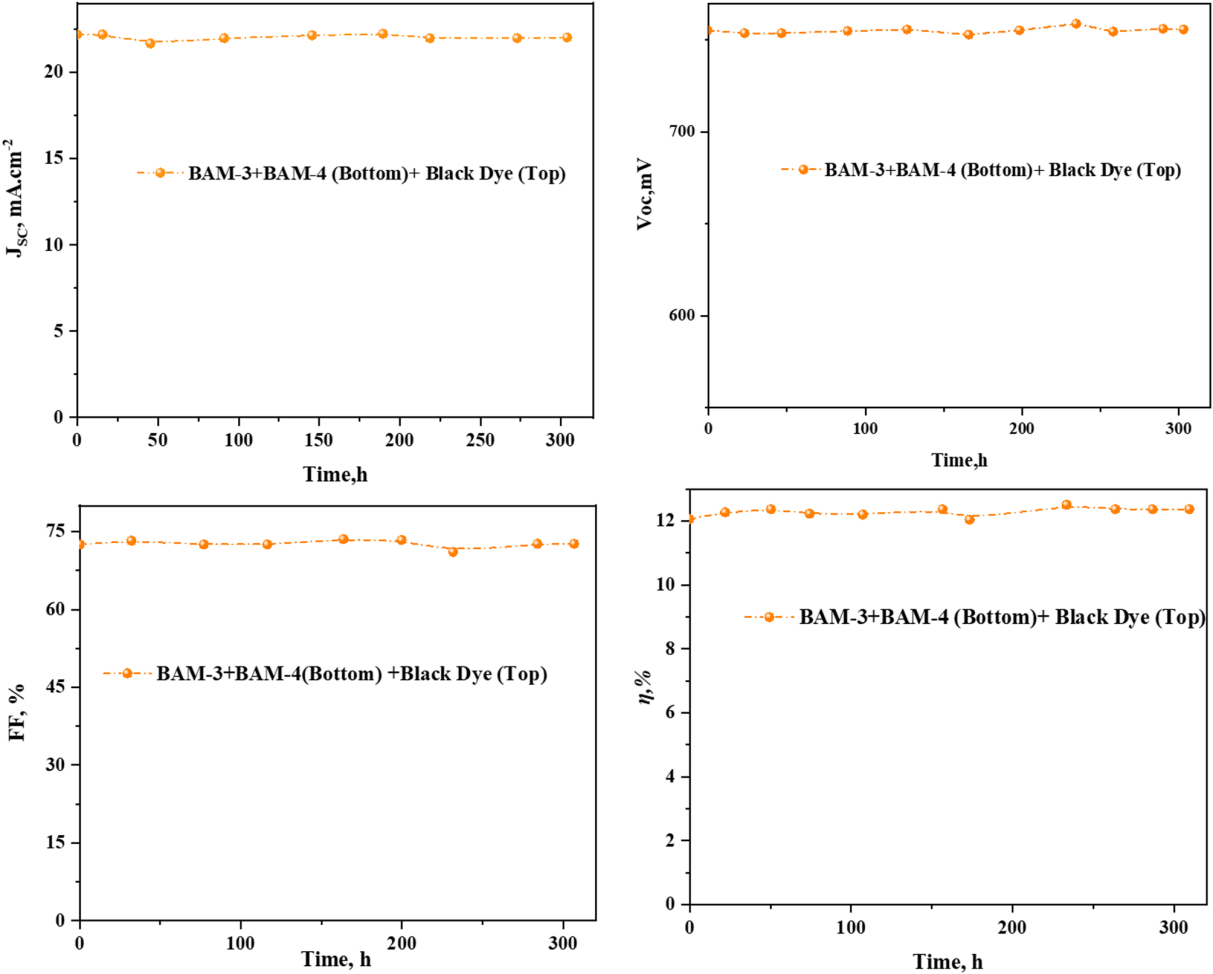
Stability of mixed PT-DSSC based on **BAM-3 + BAM-4** and **Black Dye** under illumination for 300 h.

To evaluate the long-term operational durability of (DSSCs), we monitored the photovoltaic performance of devices based on mixed (PT-DSSC) incorporating **BAM-3, BAM-4**, and Black Dye, under continuous illumination for 300 h. Key metrics, including *(V*_*OC*_*), (J*_*SC*_*), (FF),* and (*η*), were tracked over time (Fig. 14). All devices exhibited remarkable photostability, with only minimal degradation observed over the test period. Specifically, the mixed PT-DSSC system-maintained a near-constant *V*_*OC*_ with a negligible drop of less than 1% after 300 h of illumination. Similarly, both *J*_*SC*_ and* FF* remained highly stable, leading to an overall retention of more than 95% of the initial power conversion efficiency. This indicates the excellent photochemical robustness of the dye ensemble and effective suppression of charge recombination at the photoanode/electrolyte interface. This stability can be attributed to the molecular structure of the **BAM** dyes, particularly the incorporation of electron-deficient heterocycles and stabilizing substituents, which likely mitigate photooxidative degradation^41,42^. The synergistic interaction between **BAM-3, BAM-4**, and **Black Dye** appears to contribute to improved electron lifetime and interfacial charge stability, which in turn delays the onset of device fatigue.

### Structure–activity relationship (SAR) of BAM-1–4

We compare BAM dyes that share the same donor (naphthalene) and π-bridge (phenyl-pyrazole) but differ in the auxiliary acceptor. We relate steric profile, effective π-extension/planarity, and electron-withdrawing capability to (i) λ_max_, ε, E_0-0_ (Table 1), (ii) FMO/MEP/ELF distributions (Figs. 7, 8, 9), (1) Electron-withdrawing strength of the auxiliary acceptor → ICT strength, bandgap, and injection. **BAM-1** (4-nitroacetonitrile) shows the shortest λ_max_ (445 nm) and largest E0-0 (2.47 eV), indicating a wider gap and relatively weaker ICT delocalization from D → A despite the strong intrinsic -CN/-NO_2_ pull; the localized acceptor reduces conjugative communication, limiting redshift and J_SC_. **BAM-3** (4-carboxyacetamide) strengthens conjugative coupling into the acceptor, giving the largest ε and red-shift (λ_max_ 473 nm, E_0-0_ 2.29 eV) and, correspondingly, the highest *J*_*SC*_ (21.15 mA cm^−2^) and *η* (9.85%) among single devices with Black dye. **BAM-4** (pyrazolone), a 1,3-dicarbonyl-like acceptor, expands the acceptor π-system and stabilizes the LUMO; λmax 458 nm, E_0-0_ 2.33 eV and strong ε are consistent with robust ICT and broad absorption, yielding *Jsc* 20.38 mA cm^−2^, *η* 8.89%. These optical trends mirror FMO/MEP/ELF maps showing increased donor → acceptor charge delocalization from BAM-1 → BAM-4. (2) π-Conjugation & planarity vs. aggregation and interfacial recombination. Greater effective conjugation (**BAM-3, BAM-4**) correlates with higher ε, red-shifted λ_max_ (Table 1) and higher IPCE; concurrently, EIS indicates larger mid-frequency arcs (higher Rrec) for BAM-3/4 co-sensitized devices, evidencing suppressed recombination/longer lifetimes, which supports their superior Voc/FF.

Mechanistically, the phenyl-pyrazole bridge enhances rigidity; acceptor conjugation in **BAM-3/4** improves LUMO delocalization over anchoring sites (**FMO/MEP/ELF**), favoring injection and reduced back-transfer. (3) Steric hindrance around the acceptor region → aggregation control, dye loading, and FF. The bulky, out-of-plane pyrazolone ring (**BAM-4**) and amide-bearing **BAM-3** introduce steric impediments to π–π stacking, aiding monolayer dispersion and co-adsorption with Black dye; this aligns with higher R_rec_ and improved FF vs. **BAM-1/2**. Consistently, BAM-3 shows the highest dye loading (3.82 × 10^−5^ mol cm^−2^) in Table 2 and the best single-cell η. (4) Consequence for device selection and tandem behavior.

Because BAM-3/4 couple strong ICT with steric aggregation control, they pair effectively with Black dye to yield the best single-cell and PT-DSSC outcomes, including peak *IPCE* (~ 92%) and highest *PCE* (12.13%) for the tandem.

## Conclusion

In this study, we developed and characterized a new class of organic D–π–A sensitizers (**BAM-1 to BAM-4**) featuring naphthalene donor units, phenyl-pyrazole π-bridges, and acceptor groups with diverse structures. Through comprehensive optical, electrochemical, and computational analyses, we established that structural modifications to the electron-accepting moiety play a decisive role in modulating the light absorption, charge separation, and overall device performance. Among the dyes, **BAM-3** and **BAM-4** demonstrated superior light-harvesting capabilities, favorable energy alignment with TiO_2_, and high dye-loading, which translated to elevated power conversion efficiencies when co-sensitized with the **Black Dye.** The tandem configuration, which integrated **BAM-3** and **BAM-4** with Black Dye in a parallel photoanode arrangement, achieved remarkable efficiencies of 12.13% and 25.85% under standard illumination and indoor lighting (1000 lx), respectively. These improvements were further supported by enhanced recombination resistance and excellent operational stability during prolonged testing. This work provides a promising blueprint for the rational design of high-efficiency, stable sensitizers suitable for both outdoor and indoor photovoltaic applications.

## Supplementary Information

Below is the link to the electronic supplementary material.

Supplementary Material 1

## Data Availability

All data generated or analyzed during this study are included in this published article and its supplementary information files.

## Dual publication

The results/data/figures in this manuscript have not been published elsewhere, nor are they under consideration (from you or one of your Contributing Authors) by another publisher.

## Third party material

All of the material is owned by the authors and/or no permissions are required.
