# Supplementary material for: High-efficiency tandem DSSCs based on tailored naphthalene sensitizers for indoor DSSC efficiency above 25%
Source: Sci Rep. 2025 Dec 18;15:44135. doi: 10.1038/s41598-025-30854-0 (PMC12717194; doi:10.1038/s41598-025-30854-0)
Supplement: Supplementary file 1 — Supplementary Material 1 [file 41598_2025_30854_MOESM1_ESM.docx]

**High-Efficiency Tandem DSSCs Based on Tailored Naphthalene Sensitizers for Indoor DSSC Efficiency Above 25%.**

**Materials**

Alfa Aesar and Sigma Aldrich supplied the starting materials. The melting points were measured in degrees Celsius using a Gallenkamp electric melting point instrument and are uncorrected. Thermo Scientific Nicolet iS10 FTIR spectrometer was used to get IR spectra (KBr), while a Burker NMR spectrometer was used to obtain NMR spectra in DMSO-*d6* at frequencies of 400 MHz (^1^H-NMR) and 100 MHz (^13^C- NMR). A high-performance twin beam spectrophotometer (T80 series) was used to acquire UV–visible spectra. Thermo Fisher Scientific DSQ II GC/MS with Kratos MS-70 ev was used for mass analyses, while a Perkin Elmer 2400 analyzer was used for elemental analysis. The attached information file contains detailed information on the instruments and DSSC fabrications used.


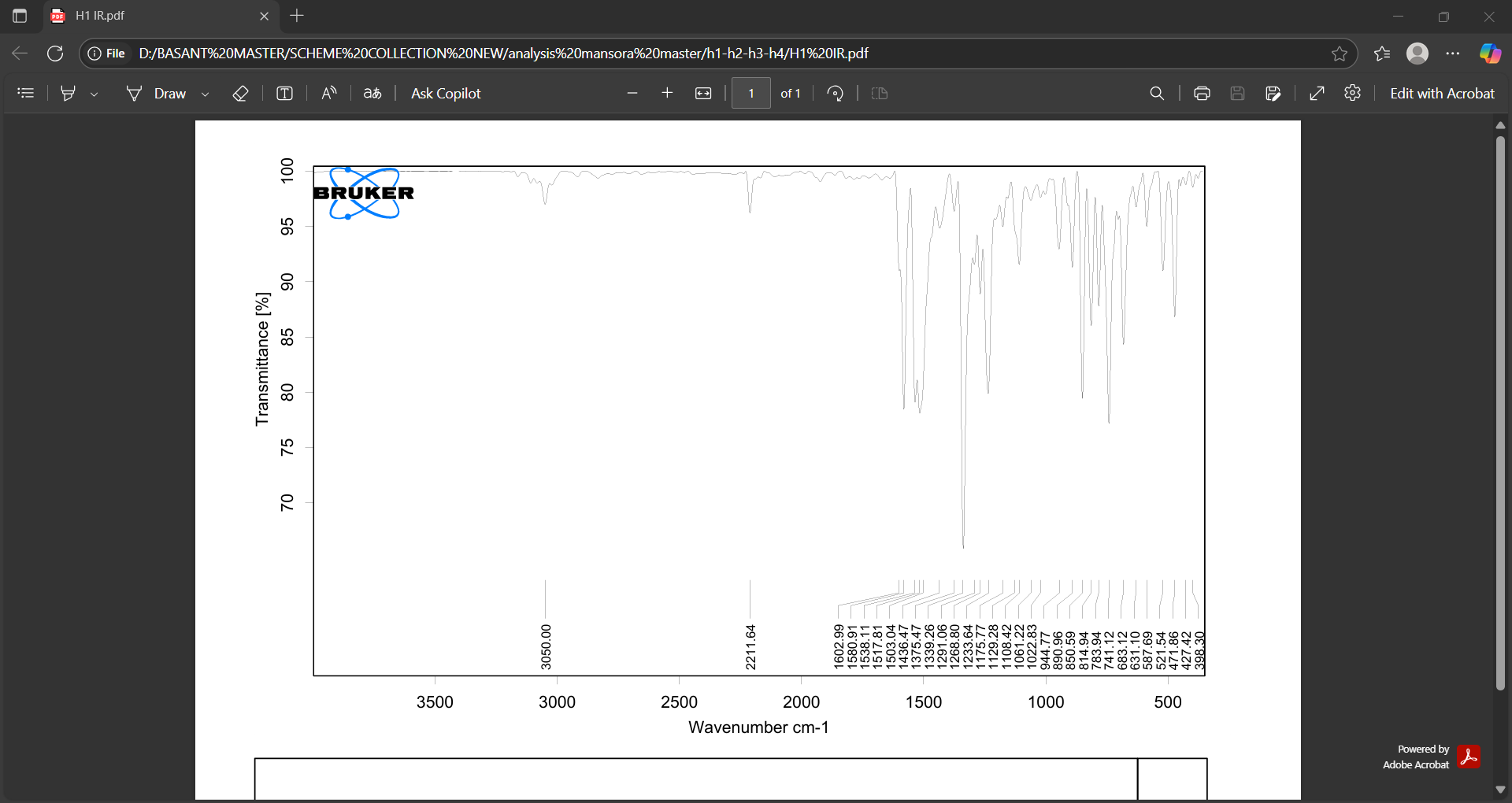


**Figure (S1): IR spectrum of compound BAM-1**

**
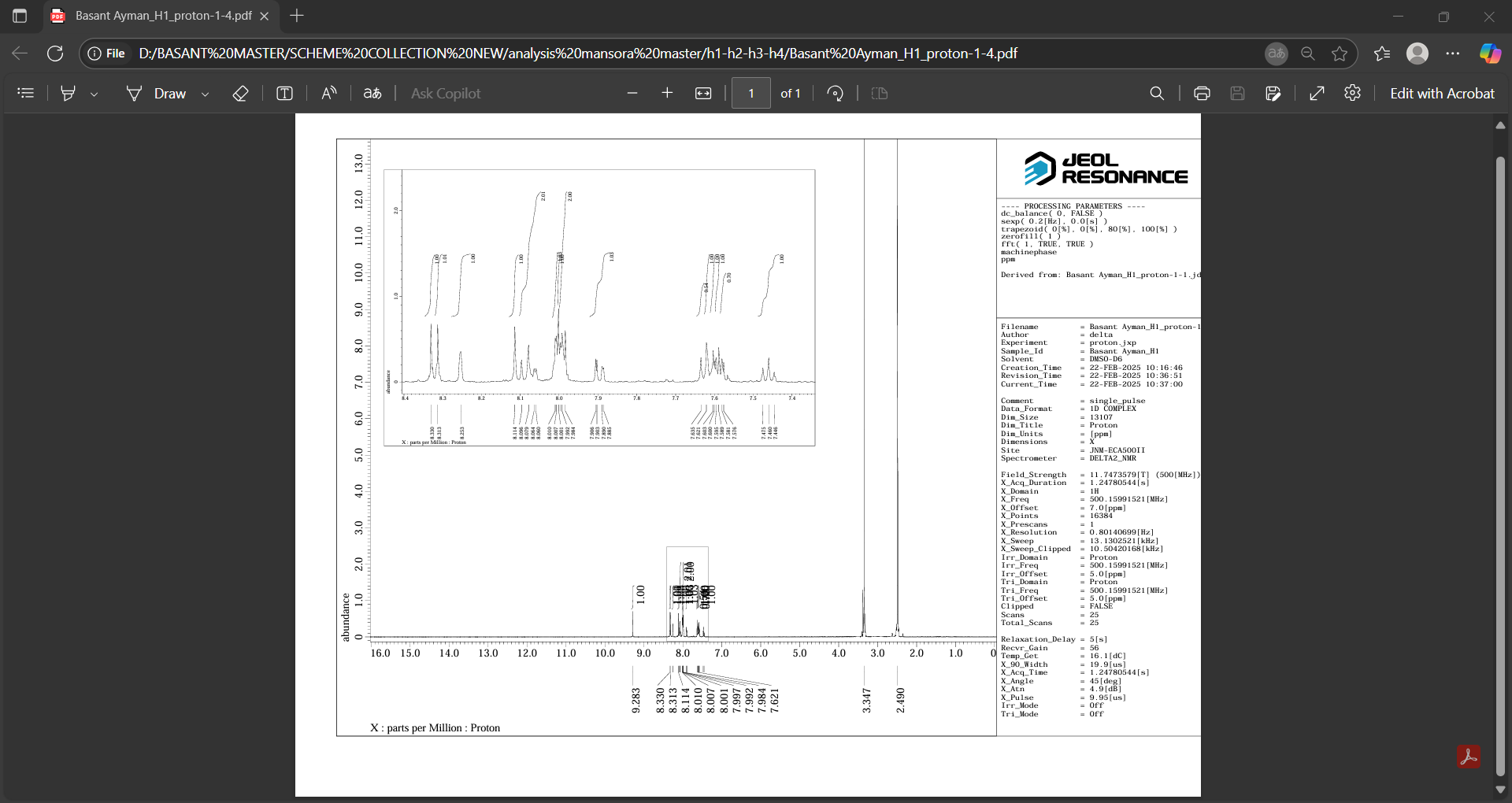
**

**Figure (S2): ^1^H NMR spectrum of compound H-1.**

**
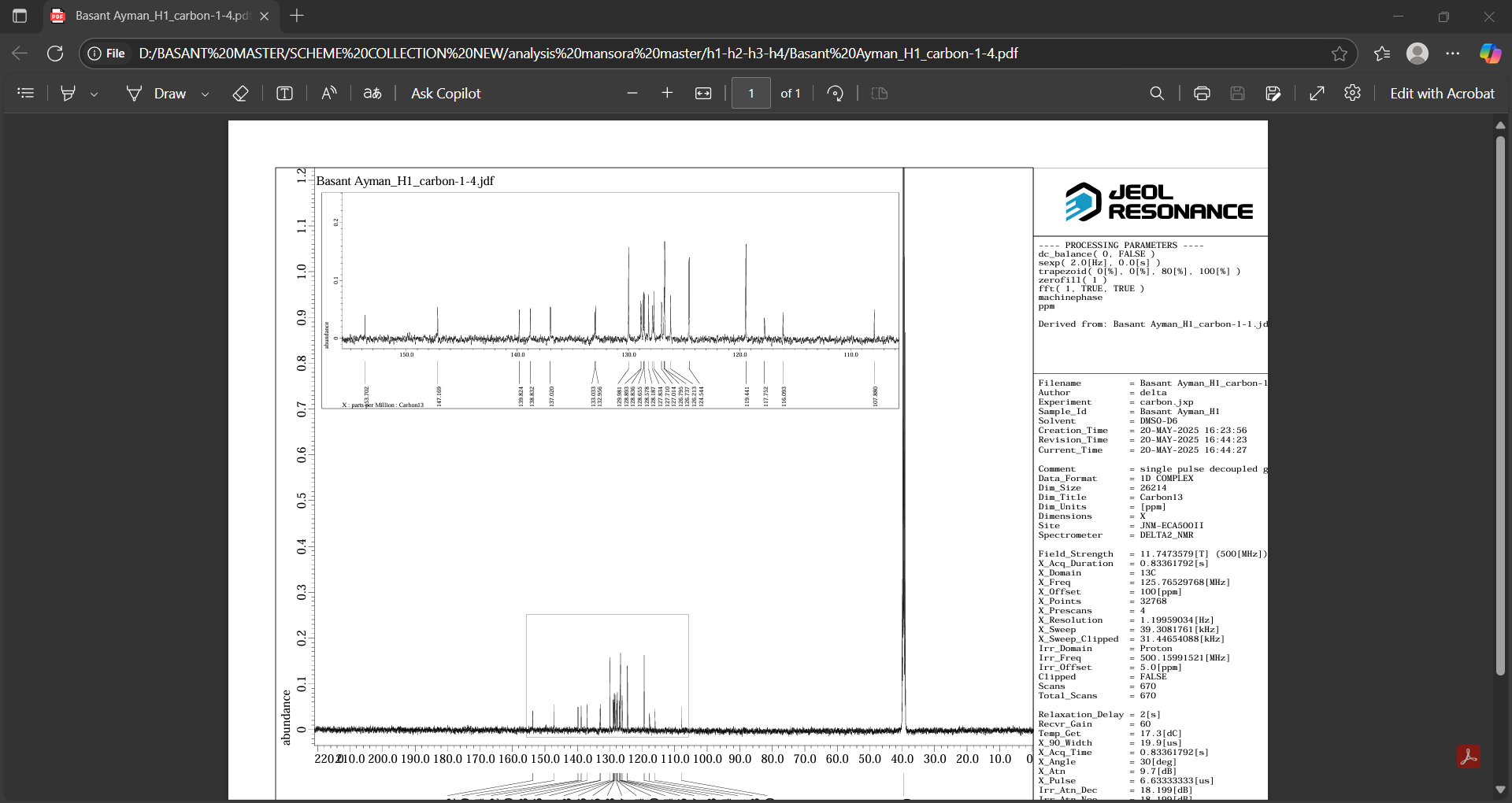
**

**Figure (S3): ^13^C NMR spectrum of compound BAM-1.**


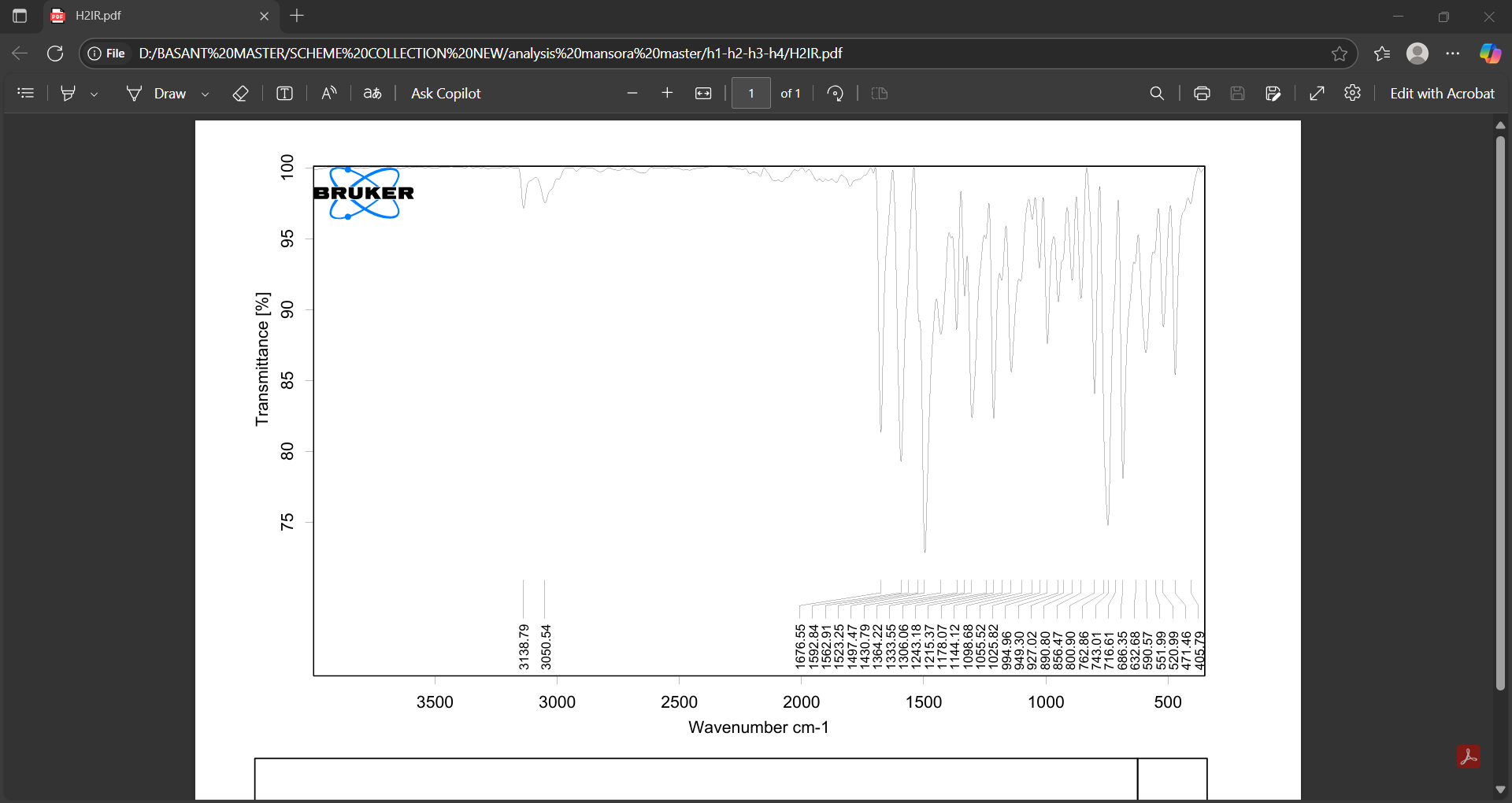


**Figure (S4): IR spectrum of compound BAM-2**


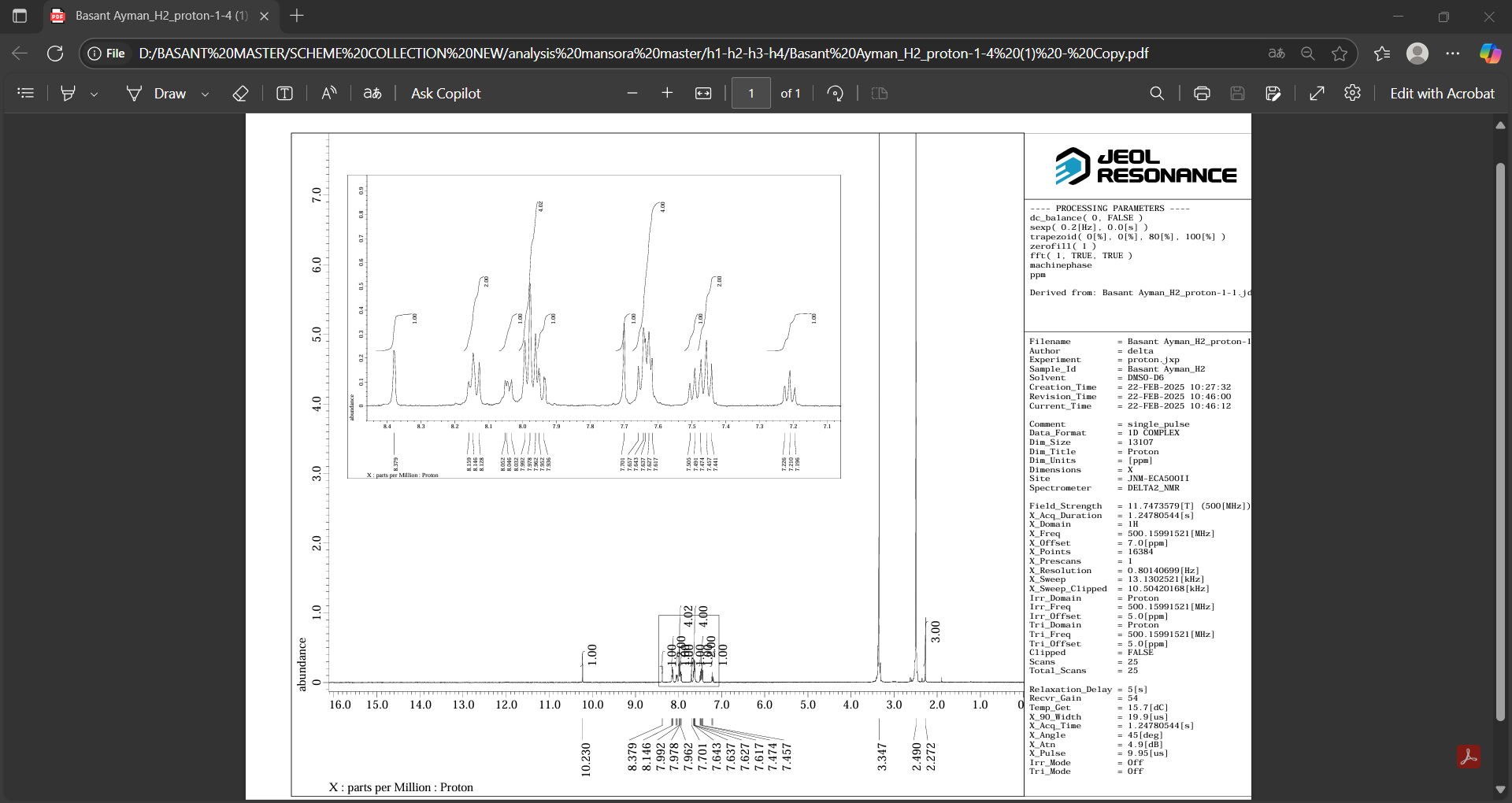


**Figure (S5): ^1^H NMR spectrum of compound BAM-2.**

**
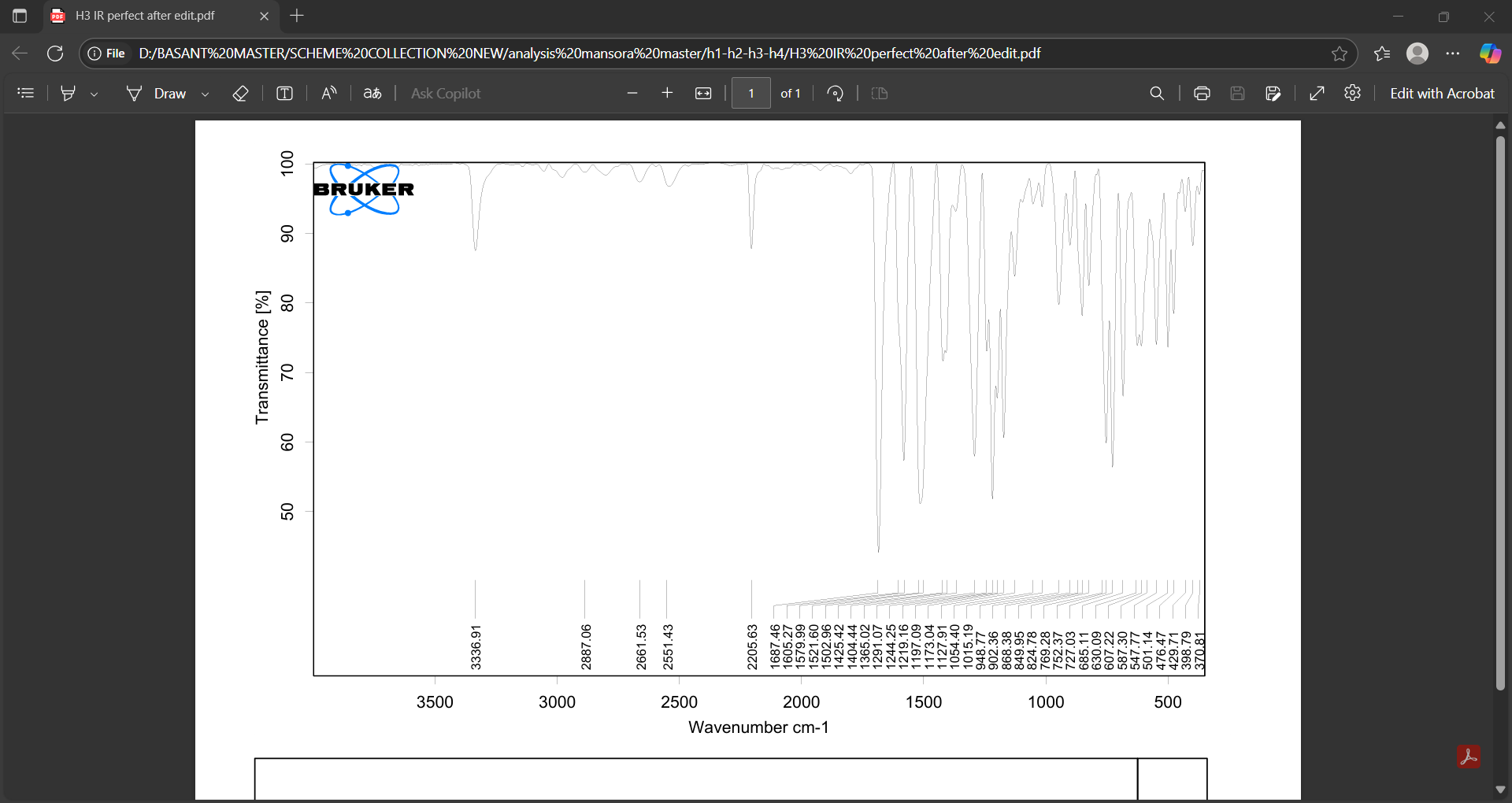
**

**Figure (S6): IR spectrum of compound BAM-3**

**
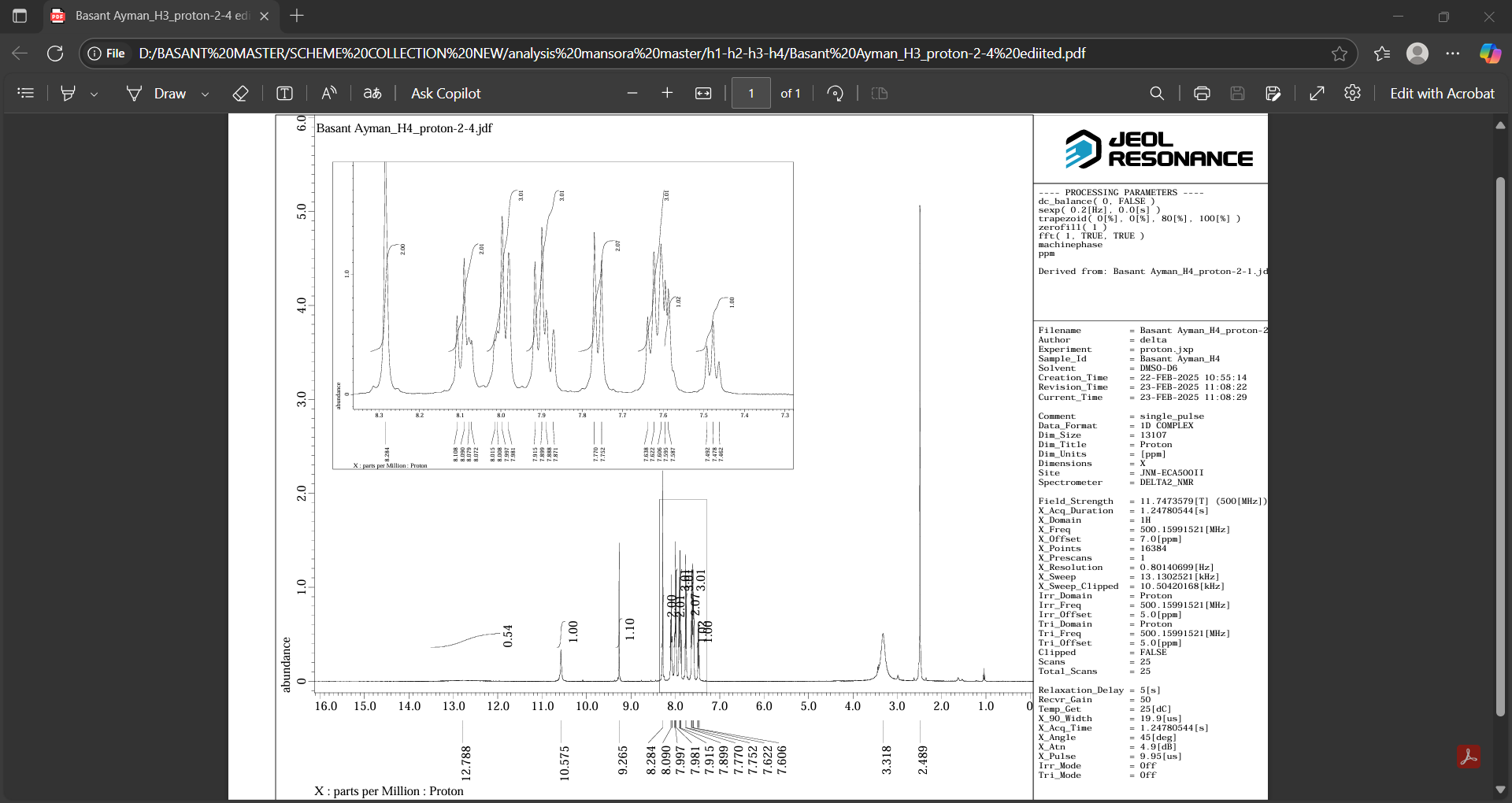
**

**Figure (S7): ^1^H NMR spectrum of compound BAM-3**


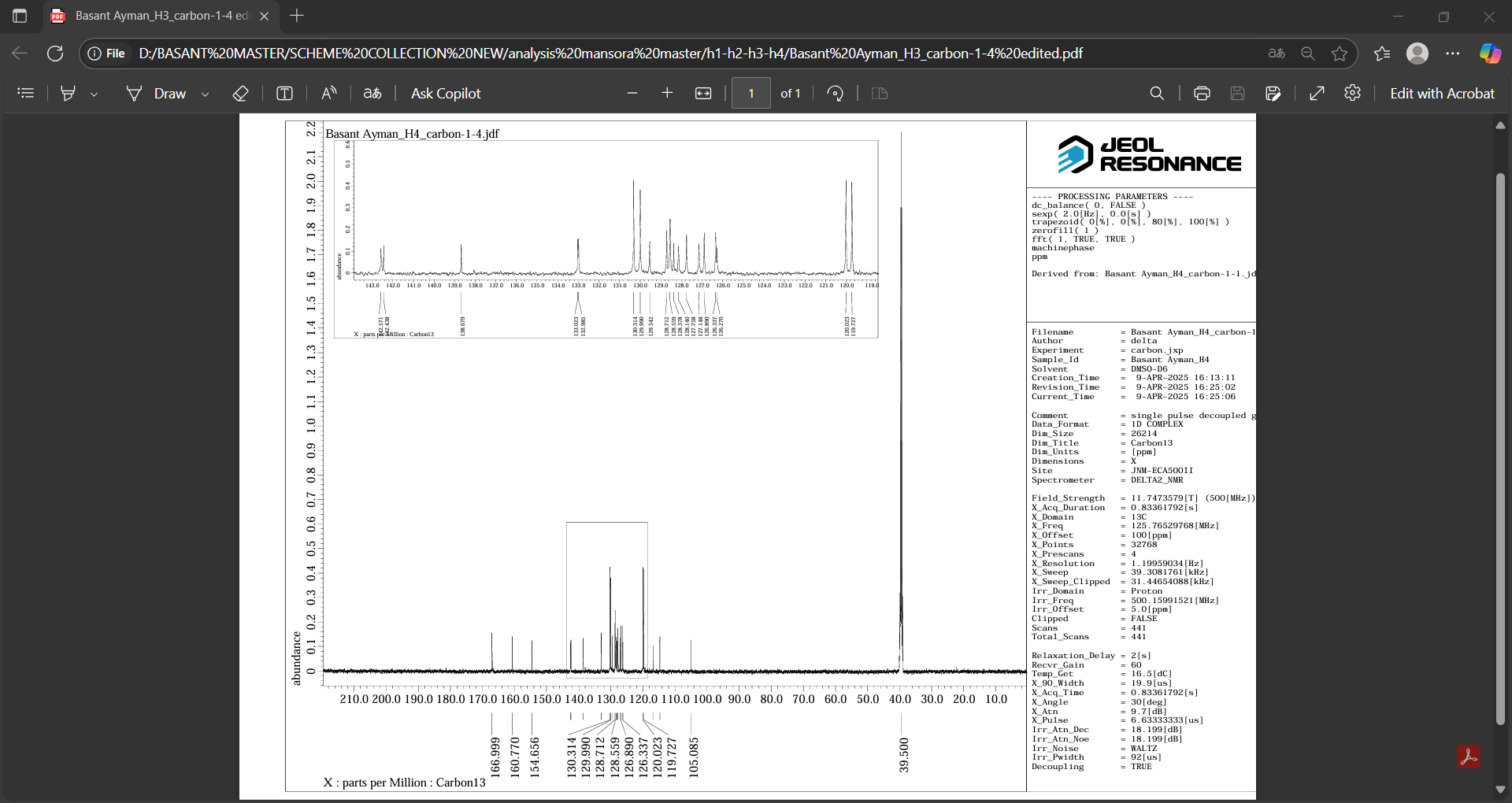


**Figure (S8): ^13^C NMR spectrum of compound BAM-3.**


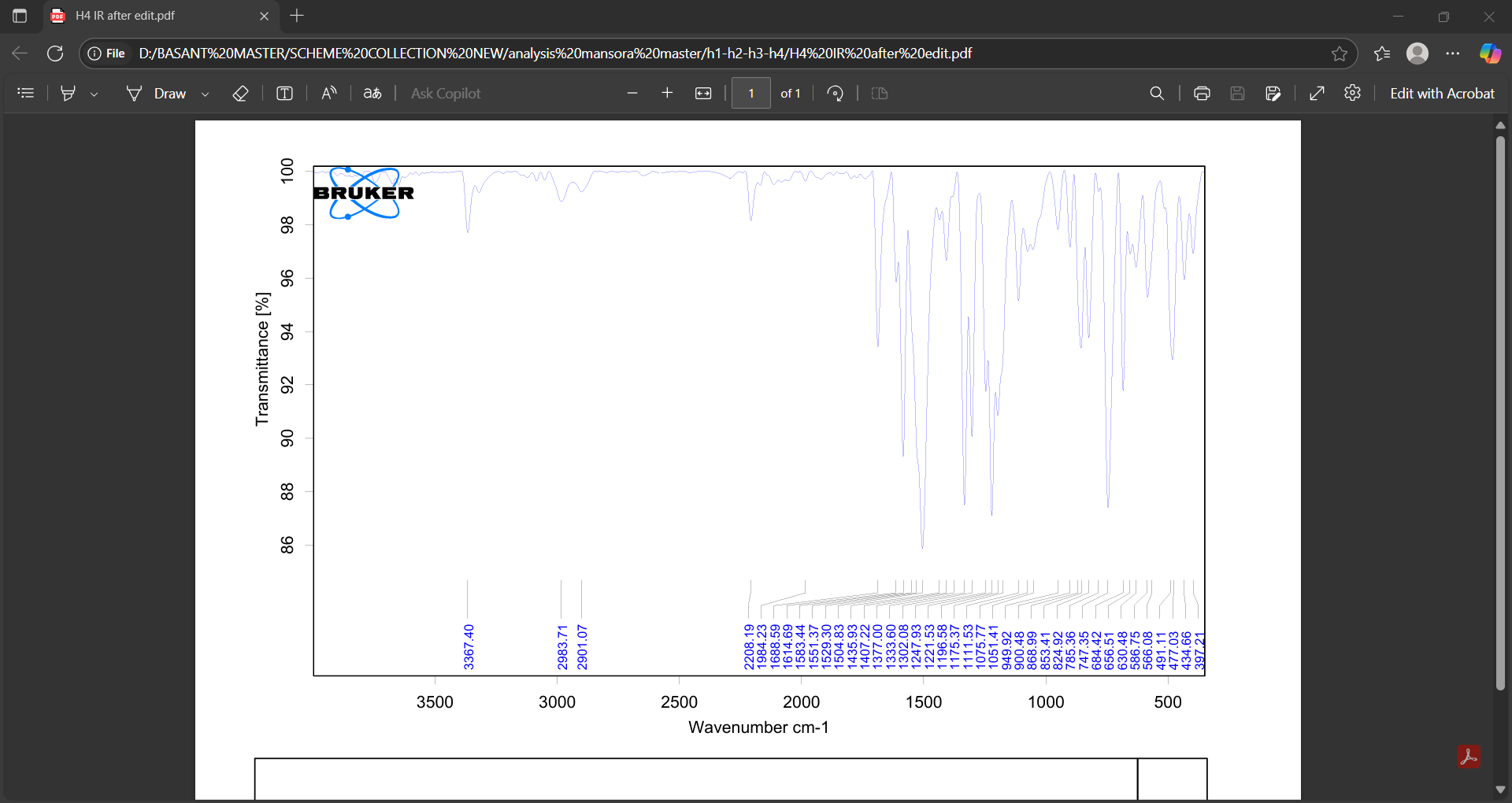


**Figure (S9): IR spectrum of compound BAM-4**


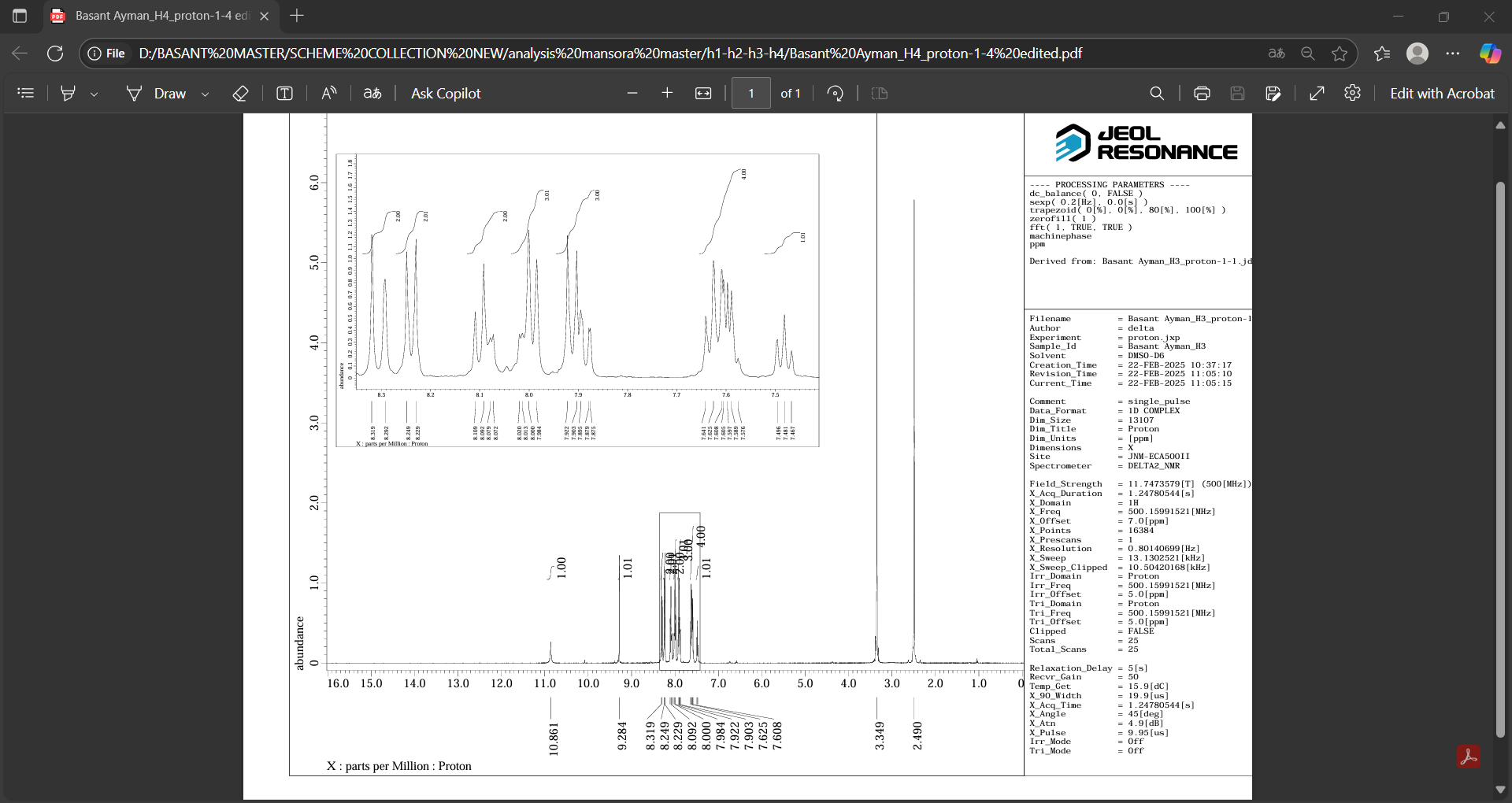


**Figure (S10): ^1^H NMR spectrum of compound BAM-4**


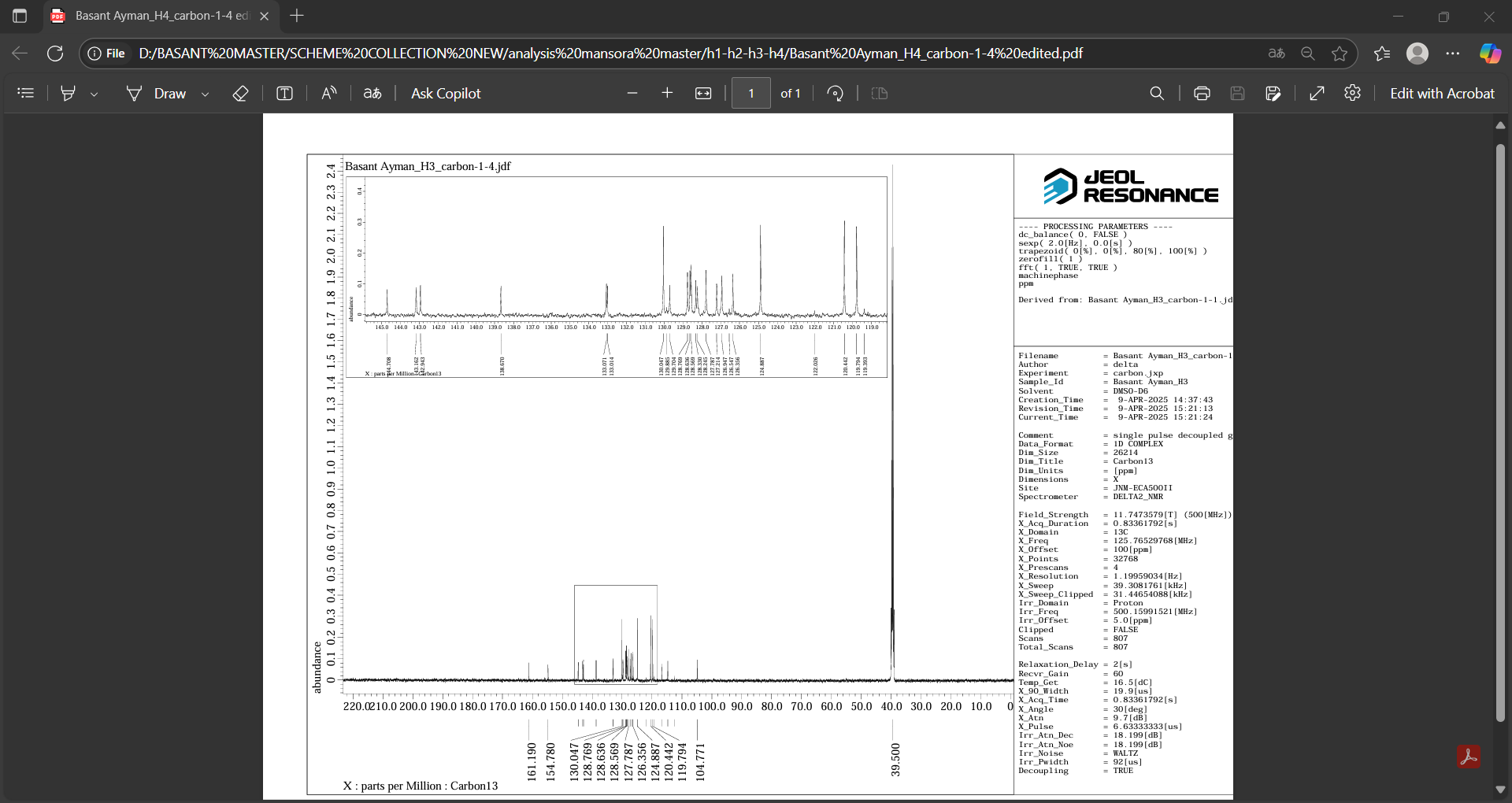


**Figure (S11): ^13^C NMR spectrum of compound BAM-4.**

**
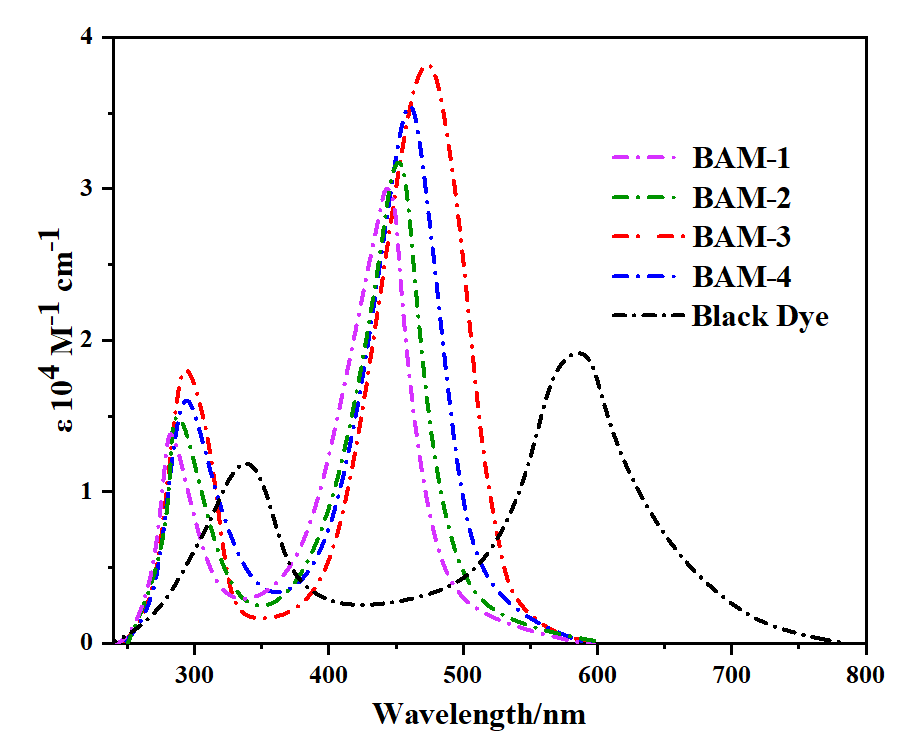
**

**Figure (S12): UV-Vis absorbation of compound BAM-1-4 and Black dye.**

**
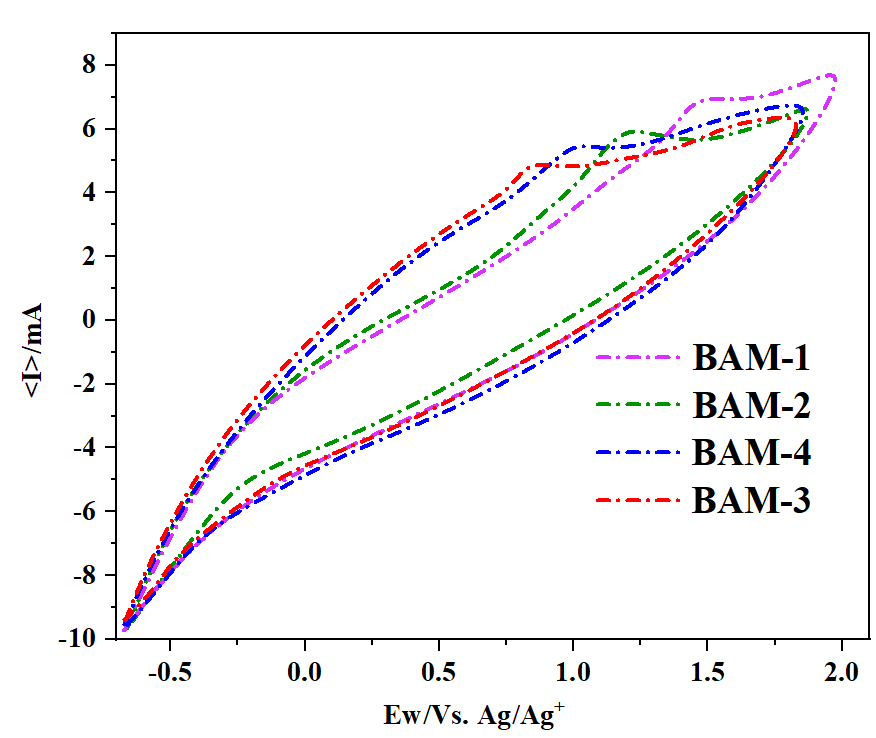
**

**Figure (S13): Cyclic voltammetry spectra of sensitizers BAM-1-4.**

**Table S1.** Compares the performance parameters of **Black dye** with recently reported high-performance sensitizer and co-sensitizer.

| **Sensitizers/Co-sensitizers** | ***V_OC_* (V)** | ***J_SC_* (mA.cm^-2^)** | ***FF* (%)** | ***η (%)*** | ***References*** |
| --- | --- | --- | --- | --- | --- |
| **(0.2 mM Black dye)** | **709** | **19.57** | **65.55** | **9.10** | **https://doi.org/10.1016/j.molliq.2024.125354** |
| **(0.2 mM MRK-1 (** | **632** | **17.32** | **45.86** | **5.02** | **https://doi.org/10.1016/j.molliq.2024.125354** |
| **(0.2 mM MRK-2(** | **667** | **18.58** | **47.44** | **5.88** | **https://doi.org/10.1016/j.molliq.2024.125354** |
| **(MRK-1 + 0.2 mM Black dye)** | **691** | **20.32** | **67.80** | **9.52** | **https://doi.org/10.1016/j.molliq.2024.125354** |
| **(MRK-2+ 0.2 mM Black dye)** | **729** | **21.45** | **63.31** | **9.90** | **https://doi.org/10.1016/j.molliq.2024.125354** |
| **Black-dye +D1** | **703** | **19.54** | **71.40** | **9.80** | [***https://doi.org/10.1155/2013/910527***](https://doi.org/10.1155/2013/910527) |
| **Black-dye+AK-01** | **685** | **20.98** | **71.10** | **10.21** | ***https://doi.org/10.1002/pssa.201431654*** |
| **Black-dye+** **U01** | **722** | **20.44** | **71.50** | **10.56** | ***https://doi.org/10.1002/pssa.201431654*** |
| **Black dye + NKX-2553** | **657** | **22.11** | **71.00** | **10.32** | ***RSC Adv., 2012, 2, 3198–3200*** |
| **Black dye + D131** | **676** | **23.23** | **70.60** | **11.08** | ***RSC Adv., 2012, 2, 3198–3200*** |
| **Black dye + D131 + DCA** | **685** | **23.34** | **70.90** | **11.33** | ***RSC Adv., 2012, 2, 3198–3200*** |

**2. Cell preparations and photovoltaic characterizations**

**2.1. Device assembly and Measurements**

The fabrication of PT-DSSCs involved several intricate steps. First, an electrolyte solution was prepared, containing 0.5 M tert-butylpyridine, 0.05 M I_2_, 0.1 M LiI, 0.1 M DMII (1,3-Dimethylimidazolium Iodide), 0.5 M BMII (1-Butyl-3-methylimidazolium Iodide), and 0.1 M guanidine thiocyanate dissolved in a mixture of acetonitrile and pentanenitrile (85:15, v/v). TiO_2_ photoanodes were fabricated by screen-printing 18NR-T and 18NR-AO pastes onto cleaned FTO glass (8 Ω/sq), followed by a programmed temperature calcination process (325°C for 10 min, 375°C for 10 min, 450°C for 25 min, 500°C for 25 min). The cooled films underwent TiCl_4_ treatment (40 mM at 70°C for 30 min) and a final calcination at 450°C for 30 min. These anodes were then sensitized with 0.2 mM solutions of **BAM-1-4** or **Black Dye** for 16 hours. Counter electrodes were fabricated by coating the FTO glasses with a thin layer of Pt by sputtering technique, the conductivity increase minimal (from 8 Ω/sq to 10 Ω/sq) and did not significantly affect the overall DSSC performance. Counter electrodes were prepared by spin-coating a 0.02 M chloroplatinic acid solution in isopropyl alcohol onto FTO glass and calcining at 600°C for 25 min. The **T-DSSC** devices were assembled by connecting top and bottom cells in parallel using double-sided Pt counter electrodes, with **BAM-3** + BAM-4 and **Black Dye** serving as the photoanode sensitizers for the top and bottom cells, respectively. J−V measurement under indoor light conditions was carried out using a custom-made setup with a warm white-fluorescent lamp/light source (Osram WWCFL, 100-1000 lux).

**Dye adsorption optimization (CDCA-free).**

All TiO_2_ photoanodes were sintered and UV-ozone treated (15 min) immediately before adsorption. Unless stated otherwise, adsorption was performed without **CDCA**. Solutions were freshly prepared, filtered (0.2 µm PTFE), and kept light protected.

- Black dye (N749) single cells. Photoanodes were immersed in 0.30 mM N749 in acetonitrile: tert-butanol (1:1, v/v) for 16 h at 25 °C, then briefly rinsed (2×, acetonitrile: tert-butanol 1:1) and N₂-dried.
- BAM dyes (single co-sensitization with Black dye). For BAM-1…4 + Black, we screened ethanol and EtOH:ACN (3:1) at 0.15–0.35 mM and 4–24 h. Optimal CDCA-free conditions used here were 0.25–0.30 mM BAM in ethanol for 10–12 h at 25 °C, followed by 0.30 mM N749 in ACN:t-BuOH (1:1) for 6–8 h (sequential co-adsorption). Gentle rinses (EtOH, then ACN:t-BuOH) and N₂ drying completed the step.
- Parallel tandem (PT-DSSC) stack. The top electrode (FTO/TiO_2_/Black) used the N749 condition above. The bottom electrode (**FTO/TiO_2_/BAM-3+BAM-4**) was prepared by sequential adsorption: 0.25 mM BAM-3 (EtOH, 10 h) → rinse → 0.25 mM BAM-4 (EtOH, 10 h); a cocktail alternative (0.15 mM + 0.15 mM in EtOH, 12 h) gave similar J_SC but slightly lower FF, so the sequential route was adopted. After adsorption, both sub-cells were assembled with the same electrolyte and allowed to soak 30 min prior to J–V/IPCE.

**Rationale and literature alignment.**

The above windows (solvent choice, 0.2–0.3 mM concentration, 8–18 h dip time) mirror commonly reported adsorption regimes in high-efficiency DSSCs; they also reflect co-sensitization workflows (sequential vs. cocktail) used to balance packing and injection without CDCA.

**Table S2**. Optimized, CDCA-free dye adsorption conditions used for devices.

| **Device** | **Adsorption mode** | **Dye solution(s)** | **Solvent** | **Conc. (mM)** | **Time (h)** | **Temp. (°C)** | **Rinse** |
| --- | --- | --- | --- | --- | --- | --- | --- |
| **Black dye** | **Single** | **N749** | **ACN:t-BuOH 1:1** | **0.30** | **16** | **25** | **ACN:t-BuOH** |
| **BAM-1 + Black** | **Sequential** | **BAM-1 → N749** | **EtOH → ACN:t-BuOH** | **0.28 → 0.30** | **12 → 6** | **25** | **EtOH; ACN:t-BuOH** |
| **BAM-2 + Black** | **Sequential** | **BAM-2 → N749** | **EtOH → ACN:t-BuOH** | **0.28 → 0.30** | **12 → 6** | **25** | **EtOH; ACN:t-BuOH** |
| **BAM-3 + Black** | **Sequential** | **BAM-3 → N749** | **EtOH → ACN:t-BuOH** | **0.25 → 0.30** | **10 → 6** | **25** | **EtOH; ACN:t-BuOH** |
| **BAM-4 + Black** | **Sequential** | **BAM-4 → N749** | **EtOH → ACN:t-BuOH** | **0.25 → 0.30** | **10 → 6** | **25** | **EtOH; ACN:t-BuOH** |
| **PT-DSSC (top)** | **Single (top)** | **N749** | **ACN:t-BuOH 1:1** | **0.30** | **16** | **25** | **ACN:t-BuOH** |
| **PT-DSSC (bottom)** | **Sequential (bottom)** | **BAM-3 → BAM-4 *(preferred)*** | **EtOH** | **0.25 → 0.25** | **10 → 10** | **25** | **EtOH** |
| **PT-DSSC (bottom, alt.)** | **Cocktail (benchmark)** | **BAM-3 + BAM-4** | **EtOH** | **0.15 + 0.15** | **12** | **25** | **EtOH** |

**2.2. Photovoltaic measurements**

**1. Size Range of the Devices and Masking Conditions:**

For all dye-sensitized solar cells (DSCs) tested, including both single-sensitizer and tandem configurations, the active area was consistently measured at approximately 0.25 cm². This uniformity ensures that all devices were evaluated under the same conditions, facilitating a fair comparison of performance metrics such as photocurrent-voltage (J–V) and IPCE. To ensure accurate and reliable *J–V* measurements, a S5 black mask with an oblong aperture of 0.0875 cm² was applied to the devices during the testing process. This mask plays a critical role in defining the working area of the cell, preventing any excess light from reaching the inactive regions or the edges of the devices, which could otherwise introduce variability in the measured current. By restricting the light to a well-defined area of 0.25 cm², we minimized edge effects and ensured that only the active region of the cell contributed to the measured photocurrent. The masking was applied uniformly across all tested solar cells, including the tandem devices. For the tandem DSSCs, which feature two photoactive layers, the mask ensured that both layers were exposed to uniform light intensity, allowing for accurate evaluation of their combined performance.

**2. Calibration and Measurement Conditions of IPCE:**

The incident photon-to-current conversion efficiency (IPCE) measurements were conducted using a QEX10 system (PV Measurements, USA), equipped with a 75 W short arc xenon lamp (UXL-75XE, USHIO, Japan) and a monochromator to selectively illuminate the cells at different wavelengths. Calibration of the incident light was performed before each measurement using a silicon photodiode (IF035, PV Measurements). This step was critical to ensure that the light intensity and spectral output of the source were accurately aligned with the reference photodiode, thereby ensuring reliable IPCE data. For tandem DSSCs, which rely on the complementary absorption profiles of the **Black Dye** and **BAM-1-4** dyes, the calibration was conducted to ensure that the illumination spectrum covered the relevant wavelength range absorbed by both dyes. The illumination spot size was carefully controlled to be slightly smaller than the active area of the test cells to avoid edge effects and overlap that could skew the measurements. This approach allowed us to assess the individual contributions of each dye in the tandem configuration and accurately capture the overall efficiency of the combined system. In the case of tandem DSSCs, the combination of **Black Dye** (which absorbs primarily in the visible range) and **BAM-1-4** (which extends absorption into the near-infrared region) required additional considerations in both the calibration and measurement stages. The tandem configuration was optimized to ensure that both the top and bottom layers were illuminated appropriately and that each layer contributed effectively to the overall photocurrent. By masking the device to 0.25 cm² and ensuring uniform light exposure across the two layers, we were able to achieve accurate measurements that reflect the true performance of the tandem devices. The electrochemical impedance spectroscopy measurements were performed to understand carrier transportation behavior and interfacial charge recombination processes in fabricated DSSCs. Currently, EIS analysis is one of the most powerful techniques used to obtain additional information, mainly interfacial reactions of photoexcited electrons in DSSCs. In the present study, EIS spectra were recorded over a frequency range of 100 mHz to 200 kHz at 298K with the Bio-Logic SP-150 impedance analyzer under the illumination under solar illumination using a solar simulator (SOL3A, Oriel) equipped with a 450 W xenon lamp (91160, Oriel). The applied voltage was set at the *V_OC_* of the DSSCs with AC amplitude fixed at 10 mV. The resultant plots were fitted *via* Z-Fit software (Bio-Logic).

**Cyclic voltammetry**

**Cyclic voltammetry (CV) was performed in DMF with the electrolyte 0.1 M [TBA][PF_6_] at a scan rate of 50 mV s^−1^. The working electrode used is the Glassy carbon, Pt wire represented the counter electrode and the reference electrode is Ag/Ag^+^ in ACN. Fc/Fc^+^ was introduced as internal reference.**

**3. Molecular Modeling**

Equilibrium molecular geometries of **BAM-1-4** calculated using the Becke's three parameter hybrid functional, Lee–Yang–Parr's gradient corrected correlation functional (B3LYP) and (6-311g(d, p)) [1-3]. The geometry optimization calculations were followed by energy calculations using time-dependent density functional theory (TD-DFT) utilizing the energy, functional B3lyp and the basis set 6-311g (d, p). The solvent (DMF) effect was accounted for by using the conductor-like polarizable continuum model (C-PCM), implemented in Gaussian 09.

**References**

[1] A. D. Becke, Phys. Rev. A **1988**, 38, 3098.

[2]C. T. Lee, W.T. Yang, R.G. Parr, Phys. Rev. B. **1988**, 37, 785

[3] N. Godbout, D.R. Salahub, J. Andzelm, E. Wimmer, Optimization of Gaussian-type basis-sets for local spin-density functional calculations .1. Boron through neon, optimization technique and validation. Can. J. Chem.-Rev. Can. Chim. **1992**, 70, 560-571.
